# Oxygen-mediated tandem polyethylene upcycling for selective aromatic synthesis

**DOI:** 10.1093/nsr/nwag207

**Published:** 2026-04-02

**Authors:** Shengming Li, Weilin Tu, Wei Zhang, Penglei Yan, Du Chen, Zhao Wang, Wenjun Chen, Panpan Xu, Mingyu Chu, Muhan Cao, Qiao Zhang, Fan Zhang, Jinxing Chen, Johannes A Lercher

**Affiliations:** State Key Laboratory of Bioinspired Interfacial Materials Science, Institute of Functional Nano & Soft Materials (FUNSOM), Soochow University, Suzhou 215123, China; State Key Laboratory of Bioinspired Interfacial Materials Science, Institute of Functional Nano & Soft Materials (FUNSOM), Soochow University, Suzhou 215123, China; State Key Laboratory of Petroleum Molecular and Process Engineering, Shanghai Key Laboratory of Green Chemistry and Chemical Processes, School of Chemistry and Molecular Engineering, East China Normal University, Shanghai 200241, China; State Key Laboratory of Bioinspired Interfacial Materials Science, Institute of Functional Nano & Soft Materials (FUNSOM), Soochow University, Suzhou 215123, China; College of Chemistry, Chemical Engineering and Materials Science, Soochow University, Suzhou 215123, China; College of Chemistry, Chemical Engineering and Materials Science, Soochow University, Suzhou 215123, China; National Engineering Laboratory of Eco-Friendly Polymeric Materials, College of Chemistry, Sichuan University, Chengdu 610064, China; Suzhou Institute of Nano-Tech and Nano-Bionics, Chinese Academy of Sciences, Suzhou 215123, China; State Key Laboratory of Bioinspired Interfacial Materials Science, Institute of Functional Nano & Soft Materials (FUNSOM), Soochow University, Suzhou 215123, China; State Key Laboratory of Bioinspired Interfacial Materials Science, Institute of Functional Nano & Soft Materials (FUNSOM), Soochow University, Suzhou 215123, China; State Key Laboratory of Bioinspired Interfacial Materials Science, Institute of Functional Nano & Soft Materials (FUNSOM), Soochow University, Suzhou 215123, China; National Engineering Laboratory of Eco-Friendly Polymeric Materials, College of Chemistry, Sichuan University, Chengdu 610064, China; State Key Laboratory of Bioinspired Interfacial Materials Science, Institute of Functional Nano & Soft Materials (FUNSOM), Soochow University, Suzhou 215123, China; Department of Chemistry and Catalysis Research Center, Technische Universität München, Garching 85747, Germany

**Keywords:** polyethylene upcycling, aerobic oxidation, aromatization, industrial grade, thermodynamic series

## Abstract

Selective chemical upcycling of waste polyethylene (PE) into high-value aromatics is conceptually very attractive. Yet, up to now the aromatic product selectivity typically remains below 50%, mainly limited by competing hydrogenolysis. We report here a cascade process that combines the selective aerobic oxidation of hydrogen, *in situ* generated from PE *via* cleavage of C(sp^3^)–H, with oxygen and the endothermic aromatization of PE. Our tandem aerobic oxidation–aromatization approach boosts the catalytic activity at moderate temperatures of up to 280°C in ambient air. Concurrently, hydrogen removal shifts the chemical equilibrium favorably toward formation of aromatic products, achieving an aromatic selectivity of 78 mol%. Demonstrated on a near kilogram scale with upscaled catalysts under mild conditions, our approach offers a scalable, energy-efficient solution for the high-selectivity conversion of plastic waste into valuable chemical intermediates and solvents.

## Teaser

By combining plastic waste breakdown with air-based oxidation, a novel process overcomes previous limits to produce high yields of aromatic chemicals at moderate temperatures.

## INTRODUCTION

The global demand for plastic products has led to reliance on fossil feedstocks to meet the requirements of synthetic plastics production [1–5]. The manufacture of plastics, however, is closely linked to significant energy consumption, carbon emissions, and waste generation. The short time of plastic products use leads to accumulation of plastic waste, posing a threat to the environment and a waste challenge to modern cities [6–8]. Notably, polyethylene (PE), among the most prevalent commodity plastics, contributes nearly 32% of the world’s plastic output, amounting to ∼120 million tons [9–13].

The catalytic deconstruction of PE typically entails the cleavage of C(sp^3^)–H and C(sp^3^)–C(sp^3^) bonds, processes that are thermodynamically limited at low temperatures [14,15]. Traditional gas phase cracking often necessitates temperatures as high as 400°C [16–20]. Recent work has successfully shown to upgrade polyolefins under mild conditions through thermodynamic coupling with exothermic reactions [21–24]. For instance, Conk *et al.* [21] combined dehydrogenation with isomerizing olefin metathesis to upcycle waste PE into monomers. Zhang *et al.* [22] have combined endothermic cleavage of the polymer C–C bonds with exothermic alkylation and isomerization, enabling full conversion of PE to liquid iso-alkanes. Selective low-temperature conversion strategies are highly desirable, as they would accelerate reactive polymer upcycling and open new avenues for decentralized catalytic upgrading.

**Figure 1.**
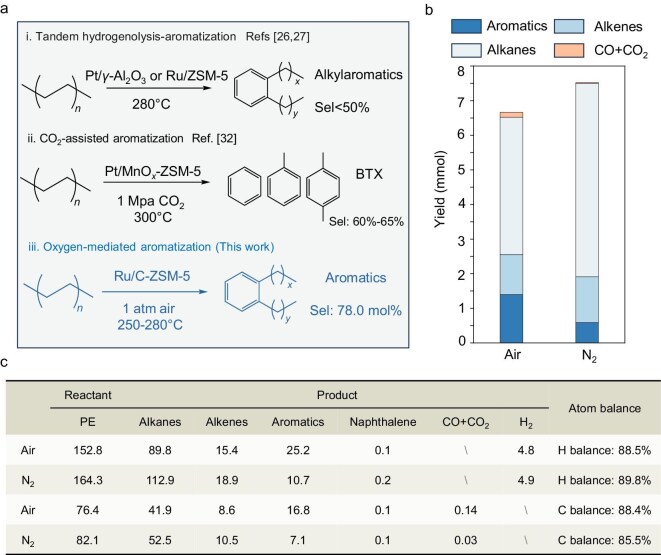
Aromatization upcycling of PE waste. (a) Upcycling of PE into aromatics by tandem hydrogenolysis/aromatization (i), tandem CO_2_ hydrogenation/aromatization (ii), and oxygen-mediated tandem PE upcycling for selective aromatic synthesis (iii). (b) The product distribution formed over the Ru/C + ZSM-5 (the ZSM-5 Si/Al ratio to 170) catalyst at 280°C for 2 h. (c) Hydrogen balance (mmol) and carbon balance (mmol) in catalytic conversion of PE for 2 h at 280°C under air/N_2_.

Combining cracking and/or hydrogenolysis with aromatization would conceptually lead to high-value aromatic hydrocarbons [25]. Zhang *et al*. [26] had reported aromatics formation first using Pt/*γ*-Al_2_O_3_, in absence of adding hydrogen producing a significant fraction of alkylbenzenes. Similarly, Du *et al*. [27] deconstructed high-density PE to aromatics in the presence of a metal and an acidic support (Fig. 1a) [26–28].

Despite various approaches to this conversion, however, the selectivity of aromatic products typically remains below 50%, primarily limited by competitive hydrogenolysis. Notably, hydrocarbon aromatization generates substantial amounts of H_2_ (C_*n*_H_2*n*+2_ → C_*n*_H_2*n*−6_ + 4H_2_), which in turn favors C–C bond hydrogenolysis over aromatization. As a result, achieving aromatic selectivity above 50% in hydrogenolysis–aromatization systems remains a formidable challenge [13,18,29].

To enhance aromatic selectivity, one potential strategy involves adding an additional reactant to consume H_2_, thereby preventing alkane formation. Several studies have reported the introduction of CO_2_ to consume hydrogen via the reverse water–gas shift (RWGS), increasing aromatic selectivity to 60%–65% [30–32]. However, the limited hydrogen capture ability of CO_2_, coupled with the strongly endothermic nature of RWGS, necessitates high reaction temperatures. Recently, Gao *et al.* demonstrated that the introduction of CO or syngas can fundamentally reconstruct the reaction pathway through CO insertion or Prins-type reactions, generating oxygen-containing intermediates, thereby promoting low-temperature aromatization and inhibiting hydrogenolysis [33,34]. An alternative approach involves introducing oxygen as a more effective H_2_ scavenger. However, it is crucial to avoid the overoxidation of PE, as this often leads to the production of oxygenated complexes, such as carboxylic acids [12,35–38], which require complicated separation processes.

Here, we present an oxygen-aided PE aromatization strategy conducted under ambient pressure using air as the oxidant at moderate temperatures of 250–280°C, achieving aromatic selectivity exceeding 78 mol%. The key conceptual advance lies in coupling endothermic C–H activation and aromatization with exothermic selective oxidation, rendering the overall process thermodynamically favorable at significantly reduced temperatures. Simultaneously, selective hydrogen removal via oxidation shifts the dehydrogenation equilibrium toward aromatics formation. Importantly, this strategy also demonstrates excellent applicability to realistic postconsumer plastics and mixed polyolefin feedstocks, maintaining high aromatic selectivity across diverse waste streams. The combination of high efficiency, operational simplicity, and feedstock robustness highlights the strong potential of this approach for scalable and practical plastic upcycling.

### Oxygen-mediated PE aromatization

In the typical oxygen-mediated aromatization procedures, a physically mixed catalyst comprising 200 mg of ZSM-5 zeolite (with a silica-to-aluminum ratio of 170, Si/Al = 170) and 50 mg of Ru/C (5 wt% Ru) was added into a flask together with commercial low-density polyethylene powder (LDPE, 2.0 g). Following a 2-h reaction period at 280°C in air, the solid conversion reached 53.5 wt%. The composition and quantity of gas products in both systems were analyzed by gas chromatography (Fig. S1). The predominant gas products were mainly C_3_–C_4_ alkanes and alkenes, with C_1_–C_2_ gases being negligible. Additionally, approximately 0.1 mmol of CO_2_ was generated, along with 0.05 mmol of CO and 0.05 mmol of H_2_. The formation of CO and CO_2_ is tentatively attributed to the excessive oxidation of hydrocarbons, while the small concentration of H_2_ originates from the dehydrogenation of LDPE. Subsequently, upon switching the atmosphere to N_2_, both the solid conversion and yield of C_1_–C_4_ hydrocarbons showed negligible change, whereas the formation of CO and CO_2_ nearly disappeared (Fig. S1).

The main components of liquid products include aromatics (alkylbenzene and naphthalene), alkenes, and alkanes (Figs S2–S4). Figure 1b illustrates the distribution of liquid products obtained in N_2_ and air, and the detailed calculation of product distribution were provided in the Note S1, Figs S5–S7, and Table S1. Approximately 1.4 mmol of aromatic molecules (selectivity ∼25.7 mol%) were generated under ambient air conditions, while only 0.59 mmol of aromatics (∼10 mol% selectivity) was detected under N_2_. The marked differences in both selectivity and aromatic yield between the two reaction systems highlight the substantial role of O_2_ in enhancing selectivity to aromatics. Additionally, carbon balance (Note S2) calculations showed that the majority of the carbon was locked into the liquid products, with only ∼3.0 mmol of carbon converted into gaseous products. The carbon and hydrogen balance (Note S3) under both atmospheres were close to 90% (Fig. 1c).

### Exploring variations

**Figure 2.**
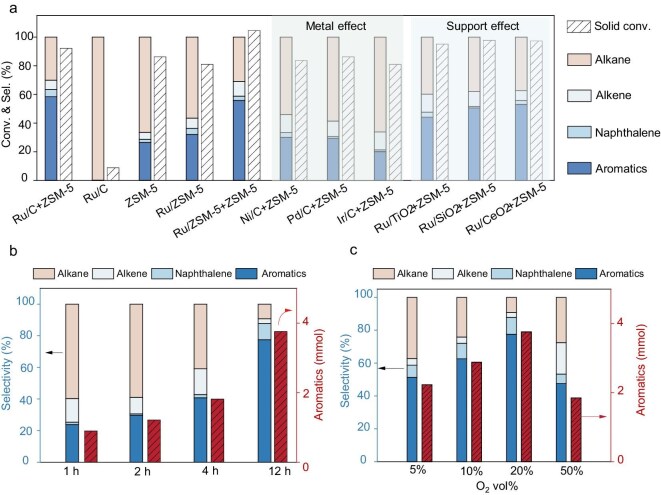
Optimization of catalytic performance. (a) Effect of metals and supports on solid conversion and product yield. Reaction conditions: *T* = 280°C, *t* = 6 h, *m*_catalyst_ = 250 mg, *m*_PE_ = 1 g (Si/Al ratio of ZSM-5: 170). Conv., conversion; Sel., selectivity. (b) Effect of reaction time on liquid product distribution over Ru/C + ZSM-5 at 280°C in air, *m*_catalyst_ = 250 mg, *m*_PE_ = 1 g (Si/Al ratio of ZSM-5: 170). (c) Effect of O_2_ concentration on liquid product distribution over Ru/C + ZSM-5 at 280°C for 12 h, *m*_catalyst_ = 250 mg, *m*_PE_ = 1 g (Si/Al ratio of ZSM-5: 170).

The oxygen-mediated PE aromatization relies on the synergistic interplay between metallic and acidic active sites. Notably, compared with the conventional metal-supported acidic catalyst (Ru/ZSM-5), the physically mixed system (Ru/C + ZSM-5, Si/Al = 170) exhibited significantly higher solid conversion (88.2 wt%) and aromatic selectivity (58.4 mol%) under identical conditions (Fig. 2a). In contrast, the supported Ru/ZSM-5 catalyst showed substantially lower activity, which can be attributed to the partial loss or masking of accessible acid sites upon metal deposition, as reflected in Table S2. Introducing additional ZSM-5 into the Ru/ZSM-5 system (Ru/ZSM-5 + ZSM-5) restored both conversion and aromatic selectivity, further confirming that direct metal deposition on the zeolite framework compromises acid functionality and limits catalytic performance. These results demonstrate that physical separation of metal and acid components better preserves acid site accessibility and enables more effective metal–acid synergy than conventional supported catalyst architectures. The nature of the metal plays a critical role by activating the C–H bonds of polyolefins. Ru, in particular, demonstrated a high C–H bond activation ability, resulting in an aromatic selectivity of 58.4 mol%, which was significantly higher compared to Ni/C (30.1 mol%), Pd/C (29.4 mol%), and Ir/C (20.1 mol%). The effect of zeolite acidity on catalytic performance was investigated by varying the Si/Al ratio to 70, 170, and 350 (Fig. S8). When the Si/Al ratio decreased from 350 to 170, the catalytic activity increased from 160 to 515 mg_PE_ h^−1^; however, when the Si/Al ratio was further reduced to 70, the activity dropped to 225 mg_PE_ h^−1^, suggesting that an optimal level of acid site concentration is required to achieve optimal synergy between the metal and the acid components. It should be noted in passing that the support had minimal influence on catalytic performance of Ru (Fig. 2a).

The impact of catalyst-to-plastic feed ratio (Fig. S9), reaction temperature (220–280°C), and time-on-stream (1–12 h) on the catalytic activity in the oxygen-mediated PE aromatization were systematically investigated (Table S3). Initial observations at 220°C for 1 h exhibited a solid conversion of 24.1 wt% with a reaction rate of 241 mg_PE_ h^−1^. Upon increasing the temperature to 250°C, a noticeable enhancement in reaction rate was observed and a large amount of aromatics was observed (Figs S10 and S11). Remarkably, after 12 h of reaction, the solid conversion reached 91.1 wt%, with a liquid aromatic hydrocarbon selectivity of 55.0 mol%. Subsequent experiments at 280°C showed a positive correlation between reaction duration and solid conversion, consistent with the trends observed at 250°C. These findings underscore the pivotal roles played by both reaction temperature and reaction time on the rate of the oxygen-mediated PE aromatization. Parameters across different temperatures, reaction times, and solid conversion rates were analyzed using polynomial regression [39–41], yielding an *r*^2^ value exceeding 0.99 (Note S4). A contour map (Fig. S10) was generated to visually elucidate observed trends.

Although the gas product distributions showed a weaker relation with reaction time (Figs S12 and S13), significant temporal changes were observed in the liquid products at 280°C (Fig. 2b and Fig. S14). In the early stages, alkanes dominated the product distribution. As the reaction proceeded, alkanes were gradually converted into alkenes and aromatics, indicative of ongoing dehydrogenation reactions. Extending the reaction to 12 h led to further conversion of alkenes into aromatics and naphthalene derivatives. Ultimately, at 280°C after 12 h, a PE solid conversion of 92.3 wt% and an aromatic selectivity of 78 mol% were achieved. To our knowledge, this represents the highest aromatic selectivity, highlighting the exceptional performance of the developed reaction systems.

Next, the influence of O_2_ concentration in the reaction atmosphere on aromatic selectivity was investigated (Fig. 2c). As the O_2_ concentration increased from 5 to 20 vol%, PE solid conversion remained steady at 85–90 wt%. While the gas-phase product distribution showed minimal variation (Figs S15 and S16), the liquid-phase distribution was highly sensitive to O_2_ concentration. Aromatic selectivity increased from 51.3 to 78.0 mol% with increasing O_2_ concentration, while alkanes decreased from 37.2 to 9.2 mol%. Thus, the introduction of O_2_ emerged as a pivotal factor influencing the liquid product selectivity in the oxygen-mediated PE aromatization. However, further increasing the O_2_ concentration to 50 vol% reduced aromatics selectivity to 47.5 mol%, and led to the formation of carboxylic acid (Fig. S17). Additionally, a notable increase in the yields of CO and CO_2_ was observed (Fig. S18), consistent with previous findings [36–38,42]. Collectively, these findings imply that excessive oxygen induces overoxidation, thereby limiting practical aromatic production.

Following the reaction, water droplets were observed in the reactor (Fig. S19), suggesting hydrogen oxidation. The reaction is concluded to shift the equilibrium for the thermodynamically challenging PE aromatization (Note S5 and Table S4). According to the Benson group increment theory, the conventional aromatization of PE is highly endothermic (Equation 1) with the Δ*G* of 15.72 kJ/mol at 280°C, indicating a thermodynamically unfavorable process at low temperatures.


(1) \begin{eqnarray*}
&&{{\mathrm{C}}}_{{n}} {{\mathrm{H}}}_{{{2n+2}}} \to {{\mathrm{C}}}_{{n}} {{\mathrm{H}}}_{{{2n-6}}}+ {\mathrm{4}} {{\mathrm{H}}}_{\mathrm{2}},\\
&&\quad \Delta {{H }} = 233.0\, {\mathrm{kJ/mol}}.
\end{eqnarray*}

To overcome this limitation, we employed a selective aerobic oxidation reaction between O_2_ and H_2_ (Equation 2), to modulate the energy landscape of the aromatization reaction. The cascading aerobic oxidation–aromatization reaction is highly exergonic. For example, at 280°C, Δ*G* = −853 kJ/mol, effectively overcoming the thermodynamic challenges associated with PE aromatization. Notably, this thermodynamic driving force also applies to the initial C–H activation step (Note S5), where selective hydrogen removal shifts the equilibrium and lowers the overall energetic barrier, as discussed above.


(2) \begin{eqnarray*}
{{\mathrm{H}}}_{\mathrm{2}}\,{+}\, 0{\mathrm{.5}}{{\mathrm{O}}}_{\mathrm{2}} \to {{\mathrm{H}}}_{\mathrm{2}}{\mathrm{O}}\left( {\mathrm{g}} \right),\,\,\,\,\Delta {{H {=} - 241}}{\mathrm{.8\,kJ/mol}}.
\end{eqnarray*}

Theoretically, to compensate for the thermal energy required for the aromatization of 1.0 g PE (∼0.0714 mol CH_2_), 0.0344 mol O_2_ needed to participate in the selective aerobic oxidation reaction, which produced 2 × 10^−5^ mol water (0.36 μL), significantly smaller than the collected amount in a batch reaction. This outcome demonstrates that the cascading aerobic oxidation–aromatization reaction is experimentally feasible, offering an effective means to enhance catalytic activity and lower the reaction temperature through kinetic coupling of the two reactions.

### Aerobic oxidation mechanism

Despite comprehensive macroscopic thermodynamic analyses clarifying the impact of O_2_ on catalytic activity and product selectivity, direct evidence of hydrogen oxidation via the interaction between surface bound oxygen and hydrogen is challenging. X-ray photoelectron spectroscopy (XPS) of spent catalysts revealed a significant increase in the valence state of Ru (Fig. S20), suggesting partial oxidation of Ru/C during the reaction. Furthermore, the combination of RuO_2_ and ZSM-5 was also active for PE aromatization (Fig. S21), with the valence state of Ru decreasing from +4 to +*δ* (0 < *δ* < 4) (Fig. S22), indicating a partial reduction of the high-valence RuO_2_. These findings show the reversibility of Ru oxidation–reduction processes, supporting the hypothesis that the lattice oxygen of the RuO_*x*_ is involved in the oxidation via a Mars–van Krevelen mechanism.

**Figure 3.**
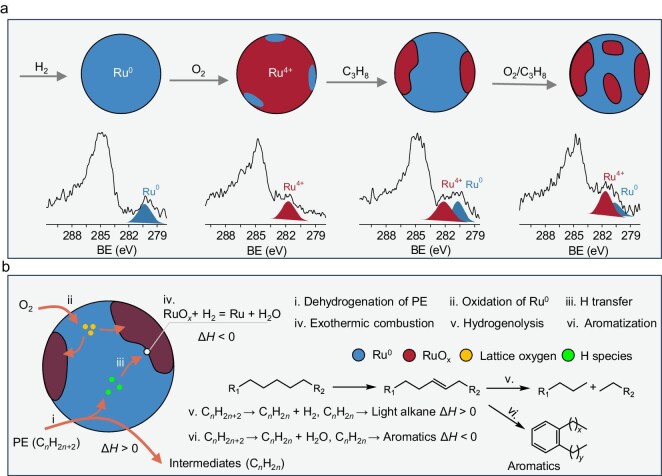
Reaction mechanism. (a) Schematic illustration of the NAP-XPS test and the corresponding Ru 3d spectra at varying conditions, *T* = 300°C, *P* = 0.2 mbar. BE, binding energy. (b) Schematic illustration of oxygen-mediated tandem PE upcycling for selective aromatic synthesis. The process is a combination of Ru hydrogenolysis, dehydrogenation, cracking aromatization in the zeolite, and removal of residual H on Ru surface.

To probe this hypothesis, *operando* near-ambient pressure X-ray photoelectron spectroscopy (NAP-XPS) was performed using Ru/SiO_2_ as a model catalyst to minimize support effects (Fig. 3a). After *in situ* H_2_ reduction, Ru exhibited a predominantly metallic state at lower binding energy. Switching to O_2_ shifted the Ru signal to higher binding energy, indicating oxidation. Upon exposure to propane (mimicking PE), the metallic Ru signal intensity increased and shifted back to lower binding energy, indicating partial reduction. When a propane–O_2_ mixture was introduced, both oxidized and metallic Ru signals remained observable, demonstrating the coexistence of Ru^4+^ and Ru^0^ under reaction conditions. Similar behavior was also confirmed by *ex situ* XPS after reaction (Fig. S23). These experiments serve to further validate the dynamic changes in the valence state of Ru as a function of the chemical potentials determined by the atmosphere composition, as summarized in Fig. 3b. After desorption from Ru, olefinic intermediates migrate into zeolite, undergoing cyclization and aromatization. Fundamentally, two pathways may (co)exist, i.e. hydrogen cleavage followed by reaction of the surface bound hydrogen with atomic oxygen and oxygen assisted hydrogen abstraction (oxidative dehydrogenation) for the initial elementary step.

### Larger-scale demonstrations

**Figure 4.**
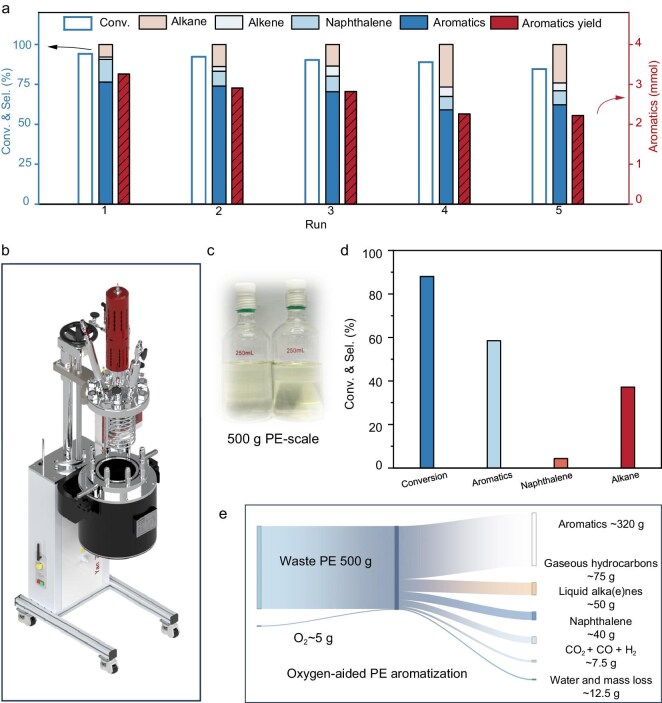
Cyclic stability and laboratory demonstration of large-scale oxygen-mediated PE aromatization. (a) The variation of the conversion rate and liquid product distributions of the oxygen-mediated PE aromatization in five cycles. Reaction conditions: *T* = 280°C, *t* = 12 h, *m*_catalyst_ = 250 mg, *m*_PE_ = 1 g (Si/Al ratio of ZSM-5: 170). (b) Schematic illustration of a reactor for the laboratory 2-L-scale oxygen-mediated PE aromatization. (c) Digital images of the liquid product. (d) The distribution of liquid products in large-scale reaction. Reaction conditions: *T* = 280°C, *t* = 12 h, *m*_catalyst_ = 25 g, *m*_PE_ = 100 g (Si/Al ratio of ZSM-5: 170). (e) Mass balance of waste PE into aromatics.

To assess the recyclability of the catalyst in the PE aromatization reaction, its reusability was systematically evaluated. After each reaction cycle, the catalyst was thoroughly washed three times with hot toluene to remove any residual unreacted PE. Remarkably, after five consecutive cycles, only minimal aggregation of the Ru nanoparticles was observed (Fig. S24), indicating good structural stability. To investigate the catalyst’s durability, the spent samples were subjected to a regeneration step involving calcination at 300°C under a H_2_/Ar (5 vol%) atmosphere for 2 h. This procedure was repeated for five cycles. Compared with the precycle, the metal content after the fifth cycle only decreased by 0.2 wt% (Table S5). As shown in Fig. 4a, the solid conversion exhibited only a slight decline, while aromatic selectivity decreased from 70 mol% to ∼60 mol%. This decrease is attributed to the partial loss of zeolite acidity (Table S6), which weakens the metal–acid synergy required for efficient cyclization and aromatization.

Notably, comparative experiments on cyclic stability were conducted in N_2_ atmosphere. Under identical conditions, the selectivity to aromatics declined from 42.7 mol% to nearly zero after three cycles (Fig. S25). Such a pronounced deterioration in performance suggests that the catalysts deactivated, which was hypothesized to be caused by acid site blocking with carbon deposits. To simulate the continuous addition of reactants, we implemented a cycle in which the reaction products were directly separated from the reaction system by distillation (Fig. S26). In this setup, 10 g of LDPE was added to the three-necked flask at 2-h intervals for each cycle, without any intervening catalyst treatment or regeneration. Figure S27 illustrates that the catalyst performance remains stable throughout three consecutive additions, indicating the suitability for continuous operation.

Building on this demonstrated stability, we further evaluated the applicability of the catalytic system using realistic waste plastics and mixed polyolefin feedstocks. As shown in Figs S28 and S29, postconsumer LDPE bags, LDPE films, high-density polyethylene (HDPE) bags, and HDPE bottles all exhibited consistently high aromatic selectivity in the range of 70.6–72.5 mol%, with complete polymer conversion. Furthermore, mixed polyolefin systems containing LDPE, HDPE, and polypropylene (PP) showed only a marginal decrease in aromatic selectivity, demonstrating strong tolerance toward feedstock heterogeneity (Figs S30–S32). These results confirm that the catalytic system maintains high efficiency across diverse and realistic plastic waste streams, highlighting its robustness under practical operating conditions.

Encouraged by the demonstrated catalyst stability and broad feedstock compatibility, we next evaluated the scalability of the process using a custom-designed 2-L mechanically stirred reactor (Fig. 4b and Fig. S33). In a single batch reaction, 100 g of plastic was used, resulting in ∼70 g liquid product, amounting to ∼350 g products within five experiments (Fig. 4c). The distribution of liquid products is depicted in Fig. 4d and Fig. S34, showcasing an aromatic selectivity of ∼60 mol%. As the reaction scale increased 100-fold, the selectivity to aromatics was only marginally reduced.

Based on large-scale experimental data, we conducted a preliminary mass balance analysis for the oxygen-mediated PE aromatization (Fig. 4e). Starting with 500 g of PE as feedstock, only about 5 g of oxygen was consumed to convert 64.0 wt% of the feedstock into aromatics (320 g), showcasing a favorable comparison with other emerging technologies for renewable arene production from biomass (Fig. S35) and CO_2_ (Fig. S36). Consequently, this newly established route presents a promising option with unparalleled efficiency for aromatics production from waste PE.

### Techno-economic analysis

A comprehensive analysis of oxygen-mediated PE aromatization for PE upcycling to aromatics through techno-economic analysis is essential [36,43–45]. We developed a model of the PE oxidation process using Aspen Plus V12 software [46]. Detailed process flow diagrams and key flow parameters are presented in Fig. S37. Furthermore, we employed the Aspen Process Economic Analyzer V12 to meticulously evaluate the capital expenditures and operating costs associated with the proposed PE oxidation plant (Table S7). Using discounted cash flow analysis, we determined the minimum selling price (MSP) for the oxidized production of aromatics from PE, defining it as the selling price at which the net present value is zero [47,48]. Finally, key process parameters were analyzed under different scenarios to account for potential uncertainties [49,50].

In the benchmark scenario, we established several key assumptions to evaluate the actual production performance of the process. First, based on a review of the literature, we assumed that the PE used for oxidation is a clean powder with a price of $0.60 per kilogram. Second, to assess the actual capacity of the process, we configured it to oxidatively depolymerize 12 500 kilograms of PE per hour, producing aromatics (C_12_H_18_) as the primary product, with hydrogen, butane, liquid alkanes, and naphthalene sold as co-products. Finally, we utilized thermal filtration to recover the catalyst for subsequent use, assuming that the catalyst is replaced six times per year.

**Figure 5.**
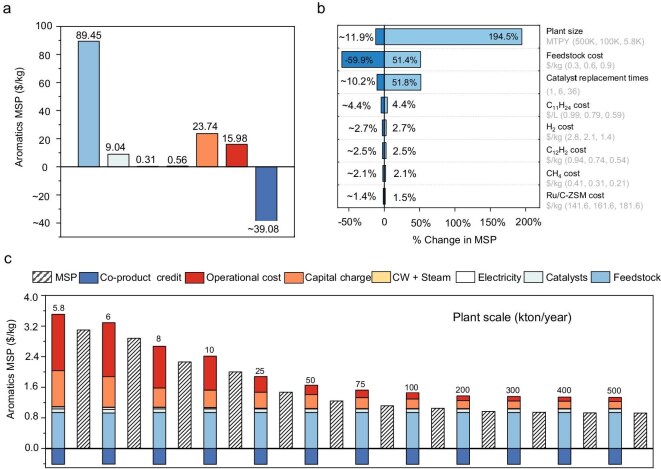
Techno-economic assessment of oxygen-mediated PE aromatization. (a) Cost breakdown of the aromatics MSP in the base case process design and as a function of PE feedstock price. (b) Tornado plot summarizing the effect of different process variables investigated on the aromatics MSP. (c) Cost breakdown of the aromatics MSP as a function of process variables of feed capacity per year. CW, cooling water.

In the base case, the MSP of aromatics was calculated to be $1.05 per kilogram, accounting for the economic contributions from by-products. As shown in Fig. S37 and Tables S8 and S9, the overall cost is dominated by the feedstock supply. Sensitivity analysis (Fig. 5a) shows that as the PE feedstock price increased from $0.30 to $0.90 per kilogram, the MSP increased from $0.42 to $1.59 per kilogram. Additional analyses of catalyst cost, replacement frequency, plant size, and by-product pricing (Figs S38–S44 and Tables S10–S17) indicate that catalyst and by-product costs have minimal impact due to the low catalyst loading and high recoverability. Furthermore, when PP was introduced as a representative impurity (Fig. S31), the MSP was calculated to be $1.038 per kilogram, indicating negligible impact on overall process economics.

By simulating the scenarios described above, we identified the key drivers influencing the MSP (Fig. 5b and Table S18). Among all variables, the feedstock cost of postconsumer PE has the most significant impact on process economics. Given the market-dependent nature of recycled plastic pricing, profitability can be further improved through plant scale-up, as shown in Fig. 5c and Fig. S45. Notably, when the annual processing capacity exceeds 8000 tons, the MSP of recycled aromatics becomes lower than the average market price of virgin aromatics ($2.08 kg^−1^), demonstrating the strong economic competitiveness and industrial potential of this air-mediated aromatization strategy.

## CONCLUSIONS

We introduce a transformative method for the selective aromatization of PE by coupling aromatization with the selective oxidation of *in situ* generated hydrogen under ambient air at atmospheric pressure, demonstrating strong engineering compatibility and potential for scalable implementation. This approach achieves aromatic selectivity as high as 78 mol%, significantly enhancing product value while simplifying downstream separation, with further improvements attainable through optimization of metal–acid synergy. Importantly, the system maintains high aromatic selectivity when applied to realistic postconsumer plastics and mixed polyolefin feedstocks, including PE/PP mixtures, highlighting its robustness toward feedstock heterogeneity. The catalyst also exhibits excellent structural stability and recyclability over multiple cycles. Nevertheless, the current system relies on noble metal active sites, and the presence of additives in real waste plastics may influence long-term catalytic performance. Future efforts toward developing non-noble metal catalysts with high activity and broad tolerance toward diverse plastic additives will be essential to further improve economic viability and long-term operational stability. Overall, this work establishes an oxygen-mediated hydrogen management paradigm for polyolefin aromatization, offering a mechanistically distinct and practically viable route for converting waste plastics into high-value aromatics under industrially relevant conditions.

## Supplementary Material

nwag207_Supplemental_File
