# Supplementary material for: Oxygen-mediated tandem polyethylene upcycling for selective aromatic synthesis
Source: Natl Sci Rev. 2026 Apr 2;13(12):nwag207. doi: 10.1093/nsr/nwag207 (PMC13317455; doi:10.1093/nsr/nwag207)
Supplement: nwag207_Supplemental_File [file nwag207_supplemental_file.pdf]

Supporting information

Oxygen-mediated tandem polyethylene upcycling for selective aromatic synthesis

Shengming Li,[1,7] *Weilin Tu,*[1,7] *Wei Zhang,*[2,*] *Penglei Yan,*[1] *Du Chen,*[3] *Zhao Wang,*[3] *Wenjun Chen,*[4] *Panpan Xu,*[5] *Mingyu Chu,*[1] *Muhan Cao,*[1] *Qiao Zhang,*[1] *Fan Zhang,*[4,*] *Jinxing Chen,*[1,*] *Johannes A. Lercher* [6,*]

[1] State Key Laboratory of Bioinspired Interfacial Materials Science, Institute of Functional Nano & Soft Materials (FUNSOM), Soochow University, Suzhou, Jiangsu 215123, China

[2] State Key Laboratory of Petroleum Molecular and Process Engineering, Shanghai Key Laboratory of Green Chemistry and Chemical Processes, School of Chemistry and Molecular Engineering, East China Normal University, Shanghai, China.

[3] College of Chemistry, Chemical Engineering and Materials Science, Soochow University, Suzhou, Jiangsu 215123, P. R. China

[4] National Engineering Laboratory of Eco-Friendly Polymeric Materials, College of Chemistry, Sichuan University, Chengdu, Sichuan 610064, P. R. China

[5] Suzhou Institute of Nano-Tech and Nano-Bionics, Chinese Academy of Sciences, Suzhou, Jiangsu 215123, P. R. China

[6] Department of Chemistry and Catalysis Research Center, Technische Universität München, Lichtenbergstr. 4, Garching, Germany

[7] These authors contributed equally to this work

Contents

Experimental sections

Supplementary Notes 1-5

Supplementary Figures 1-45

Supplementary Tables 1-18

Experimental section

Materials. $RuCl_3·3H_2O$ (Ru 36.5 wt%) and 1,4-dioxane (99%) were purchased from Bidex Pharmaceuticals company. SiO_2 and ZSM-5, (Si/Al (70, 170, 350)) purchased from Pioneer Nano corporation. Iridium Carbon (Ir/C, Ir 5 wt%), Palladium carbon (Pd/C, Pd 5 wt%), Ruthenium carbon (Ru/C, Ru 5 wt%), Nickel carbon (Ni/C, Ni 5 wt%), Methyl sulfoxide-d6 (C_2D_6OS) and 1,1,2,2-tetrachloroethane-d_2 ($C_2D_2Cl_4$) purchased from Macklin Company. Polyolefin materials of low-density polyethylene (LDPE, M_w = 4000, M_n = 1700) was purchased from Sigma-Aldrich.

Characterizations. The catalytic liquid products were quantitatively analyzed by Nuclear magnetic resonance spectrometer (NMR, AVANCE NEO 400) and GC-MS (8860-5977C). The collected liquid is filtered through a filter head, and 20 μL of the original liquid is added to a standard ($C_2D_2Cl_4$) for testing. Another method is suitable for the case of low liquid content, the liquid product is extracted with a small amount of 1,4-dioxane, and then 20 μL of liquid is mixed with a standard (1,4-dioxane or C_2D_6OS) for subsequent testing. Liquid product number average molecular weight (M_w) by Gel Permeation Chromatography (GPC, Column oven: Ht-330) test, mobile phase is tetrahydrofuran, according to the proportion of liquid concentration at least 2 mg/mL for testing. The gas products were collected using a standard 0.3 L bag and analyzed by injecting 0.5 mL of the gas into a gas chromatograph (GC-7900) for result determination. The images of scanning transmission electron microscopy (STEM), transmission electron microscopy (TEM) and corresponding elemental mapping were performed by the field emission high-resolution transmission electron microscopy (FEI Talos F200X, Thermo Fisher). The as-obtained samples were dispersed in the ethanol solution, subsequently added dropwise onto the Cu grids of 400 mesh (Electron Microscopy Sciences) for the TEM and STEM tests. The acid testing of catalysts using pyridine infrared (Bruker Tensor 27, Germany). NAP-XPS data were collected at UHV at 25 ^oC and then heat treated with a laser at 300 ^oC for 60 min in a pure hydrogen atmosphere (0.2 mbar). Then the atmosphere was switched to O_2, heated to 300 ^oC and maintained for 60 min, and then propane gas was introduced and maintained at 300 ^oC for 60 min. After the end of the first stage reaction, H_2 was used to continue the reduction at 300 ^oC for 60 min, and then propane: O_2 (4:1) ratio gas was introduced for 60 min. The C 1s peak of 284.4 eV and O 1s peak of 531.1 eV were used to calibrate the binding energy.

Catalyst preparation. ZSM-5 zeolite was prepared by calcination at 500 °C for 2 h before reaction. The Ru/C catalyst was calcined for 2 h in a tube furnace at 5% H_2/Ar at 300 °C for the reaction. The two pre-treated catalysts are physically mixed and then ground for subsequent reaction. In a typical preparation for Ru-SiO_2 (required for Nap-XPS), $RuCl_3·xH_2O$ powder was added to the deionized water to homogenize the SiO_2 powder with ultrasonic, then it was placed on the mixing table and stirred and heated. After the solvent volatilized, it was put into a vacuum oven and dried overnight at 60 ^oC. Then the catalyst was ground and then heated in a tube furnace at an acceleration rate of 10 ^oC/min for 2 hours at 300 ^oC.

Catalysis in the reactor system. The oxygen-mediated aromatization of polyolefin was carried out in a 50 mL or 2 L (large-scale experiment) stainless steel autoclave (YZPR-50(M), YZHPR-2000 (M), Shanghai Yanzheng Experimental instrument Co., Ltd.). A physical powder mixture containing the catalyst and polyolefin is placed at the bottom of the reactor. Before the reaction begins, the autoclave goes through a series of steps: it is purged at least five times with oxygen of different oxygen levels or pure Air, maintaining conditions of normal pressure. After the reaction is completed and cooled to room temperature, the gaseous product is collected in a gas sampling bag. After the liquid is extracted, the liquid/solid phase product is dissolved/dispersed in toluene and refrigerated at 4-6 ^{o}C. The liquid product is separated by centrifugal separation and the solid residue is dried overnight in an oven at 90 ^{o}C. The solid conversion rate formula of the reaction is as follows:

$$Solid\ Conv.(\%) = \left(1 - \frac{m(solid\ residuals)\ -\ m(catalyst)}{m(plastic)}\right) \times 100\%$$

Catalysis in the three-neck flask system. The oxygen-mediated aromatization of polyolefin was carried out in a 100 mL three-necked flask and placed in a heating jacket. A thermocouple is placed on the left side of the bottle mouth and inserted under the plastic powder, a condensing tube is placed in the middle of the bottle mouth, and a gas pipe is placed on the right side of the bottle mouth and inserted into a 20 mL glass bottle (glass bottle as ice bath) for collecting liquid. After the reaction, it is dissolved with dichloromethane and stored at 4-6 ^{o}C. The liquid product is separated by centrifugal separation and the solid residue is dried overnight in an oven at 90 ^{o}C.

Reusability test. The cycle test was carried out using standard procedures. The purification process is outlined as follows: the purified catalyst and product mixture is collected in a 20 mL glass bottle and 5 mL of toluene is added. The solution is stirred for 10 min, during which the remaining solid hydrocarbons dissolve in the hot toluene and the catalyst settles to the bottom of the glass bottle. The toluene containing dissolved hydrocarbons is removed while it is still hot, leaving a precipitated catalyst at the bottom. This process is repeated five times to ensure the complete removal of hydrocarbons attached to the catalyst surface. The waste catalyst was separated by centrifugal separation. Subsequently, the remaining catalyst was dried and recovered in the oven, and then reduced by a tube furnace at 300 ^{o}C for 2 h after drying. Typically, each repetition results in a catalyst loss of about 20%. In the following LDPE aromatization reaction, the catalyst consists of recovered catalyst and part of fresh catalyst. The main cause of catalyst loss is that some of the catalyst sticks to the agitator or reactor wall during the recovery process. After scaling up the reaction, we observed a significant reduction in the loss ratio. For example, when the amount of catalyst is 500 mg, the catalyst recovery rate can reach about 95% when recycled 5 times.

Supplementary Note 1. Determination and calculation of liquid product distribution

The complexity of the 1H NMR spectra arises from the presence of multiple isomers, yet their integration across various functional group regions allows for a comprehensive analysis.[1–3] Subsequent calculations involved studying the 1,4-dioxane or dichloromethane soluble liquid hydrocarbons recovered from the high pressure reactor or three-necked flask after depolymerization of PE (Mw = 4000).

Analysis of the ^{1}H NMR spectrum (Supplementary Figs. S3-S4) categorizes the product primarily into four types: aromatics (alkylbenzenes, alkylnaphthalenes), long-chain alkenes, and long-chain saturated alkanes. Due to the complexity and stochastic nature of polyolefin reactions, precise analysis of products is challenging. However, through analysis of proton nuclear magnetic resonance (NMR) spectra, we can roughly confirm the major types of aromatic hydrocarbons present (Supplementary Figs. S5-S6). Quantitative integration using NMR tools revealed an $H_a/H_\alpha(-CH_2)$ ratio to 1.1, indicating that the aromatic hydrocarbons in the products are predominantly disubstituted rather than monosubstituted. This hypothesis was further supported by the results of GC-MS analysis (Supplementary Fig. S7). Specifically, structures with chemical shifts in the range of 6.8-7.5 and 2.0-2.5 (H_α) are classified as alkylbenzenes with a single benzene ring, denoted as H_{mono}. Chemical shifts between 7.5-8.3, indicating two benzene rings, are classified as alkylnaphthalenes, denoted as H_{poly}. Additionally, regions with chemical shifts between 4.9-5.5 are assigned to long-chain alkene structures, hypothesized to possess two double bonds, labeled as H_{alkene}.

In a standard liquid-phase selective calculation conducted over a 2-hour air reaction, the H_{mono} to H_{alkene} ratio is 1.85. Consequently, the molar ratio of alkylbenzenes (each with 4H) to dialkene (each with 4H) is also 1.85. Similarly, the H_{poly} to H_{alkene} ratio is 0.02, resulting in a molar ratio of alkylnaphthalenes (each with 6 H) to dialkene (each with 4 H) of 0.03. If dialkene represents x mol% of all products, alkylbenzenes constitute 1.85x mol%, alkylnaphthalenes represent 0.03x mol%, and the remaining fraction represents saturated alkanes (1-2.88x) mol%. According to GPC analysis (**Table S1**), the liquid hydrocarbon products have an Mn value of 168 g mol^{-1} (corresponding to an averaged carbon number C_{12}). The aromatic protons ($H_{Aromatics}$) in the products are distributed among alkylnaphthalenes (0.18x mol%) and alkylbenzenes (7.4x mol%). Furthermore, the total protons (H_{total}) are distributed among alkylnaphthalenes ($C_{12}H_{12}$, 12×0.03x mol%), alkylbenzenes ($C_{12}H_{18}$, 18×1.85x mol%), dialkene ($C_{12}H_{22}$, 22x mol%), and saturated alkanes ($C_{12}H_{26}$, 26×(100–2.88x) mol%). The ratio H_{Ar}/H_{total} is given by eq S1:

$$\frac{H_{ar}}{H_{total}} = \frac{7.4x+0.18x}{18\times1.85x+12\times0.03x+22x+26\,(100-2.88x)} = 0.045 \qquad \text{(S1)}$$

The resulting dialkene mole fraction is x = 13.9, while the alkylbenzene mole fraction is 1.85x = 25.7 and alkylnaphthalenes mole fraction is 0.03x = 0.4, the saturated alkane mole fraction is 100-

2.88x = 60. Therefore, the overall selectivity to aromatics in the 1,4-dioxane-soluble hydrocarbons is 26.1 mol% in 2-hour air reaction.

Supplementary Note 2. Carbon Balance

A typical carbon balance calculation is mainly carried out according to the following steps. The total molar number of carbon atoms in 2 grams of polyethylene is 143 mmol. Under air conditions, the reaction is carried out at 280 degrees Celsius for 2 hours, with a typical conversion rate of about 53.5% and a carbon molar number ($C_{conv.}$) of 76.4 mmol. After the catalytic reaction, the main destinations of the plastic are the gas phase and the liquid phase, as well as some partially insoluble substances. In the gas phase, the destination of carbon mainly includes C_1-C_4, as well as CO_2 and CO. In the liquid phase, the destination of carbon is mainly alkanes, alkenes and aromatics with an average carbon number of 12, as well as a small amount of alkylnaphthalene. By multiplying the molar number of the product by its corresponding carbon number, we can get:

$$C_{total} = n_{alkanes} + n_{alkenes} + n_{aromatics} + n_{naphthalene} + n_{CO_x} = 67.5\ mmol$$

$$Carbon\ balance = \frac{C_{total}}{C_{conv.}} \times 100\% = 88.4\%$$

There is a percentage of missing carbon, which may be caused by the products mixed with catalyst and loss in reactor.

Supplementary Note 3. Hydrogen Balance

The number of hydrogen atoms in 2 g of polyethylene is 286 mmol. Typically, under air conditions, the solid conversion rate at 280 ^{o}C for 2 hours is approximately 53.5%, suggesting a total hydrogen release (H_{total}) of 152.8 mmol. Similar to the calculation of the carbon balance (Supplementary Note 2), regarding the gas phase, the gases associated with hydrogen are C_1-C_4 and H_2. In the liquid phase, it is mainly H on various hydrocarbon substances. Multiplying the number of moles of the product by the number of hydrogen atoms it corresponds to gives:

$$H_{total} = n_{alkanes} + n_{alkenes} + n_{aromatics} + n_{naphthalene} + n_{H_2} = 135.3\ mmo$$

$$Hyrogen\ balance = \frac{H_{total}}{H_{Conv.}} \times 100\% = 88.5\%$$

Noteworthily, nearly 10% of the loss can be attributed to water that cannot be accurately quantified.

Supplementary Note 4. Regression Analysis

Polynomials were employed to model the relationship between reaction time, temperature, and solid conversion rate, utilizing a methodology rooted in Scheffé's statistics of mixtures principles.[4,5] Originally developed, the statistics of mixtures model empirically fits the properties of a mixture by employing a polynomial to represent both linear and higher-order contributions from each component. An implicit assumption in this model posits that the sum of the weight, mole, or volume fractions of each component ($x_1+x_2+x_3+ ... +x_n$) equals 1. Despite this assumption being disregarded for the purposes of this study, the representations of the component contributions were retained, as they were deemed relevant from both chemical and mathematical perspectives.

For the case of two independent variables, the general fitting polynomial adopted was $y=a_1x_1+a_2x_2+a_{12}x_1x_2+b_1x_1(x_1-x_2)+b_2x_2(x_1-x_2)$. In this equation, x_1 represents temperature in °C, and x_2 represents time in hours. The a_nx_n terms signify the linear contributions to y, while the other terms denote the contributions of interactions between time and temperature. The linear terms, $a_1x_1+a_2x_2$, consistently featured in the polynomials. A Visual Basic for Applications algorithm in Microsoft Excel facilitated a search for candidate polynomials. Robust linear regression, incorporating Tukey's iterative, biweight function with a tuning constant of 6, was employed to minimize the impact of noise in the data.[6,7]The goodness-of-fit, r^2, was calculated using the expressions provided in equations 1−3.

$$TSS = \sum y^2 \tag{1}$$

$$RSS = \sum(\hat{y} - y)^2 \tag{2}$$

$$r^2 = \frac{TSS-RSS}{TSS} \tag{3}$$

where TSS is the total sum of squares, y is the experimentally observed value, RSS is the residual sum of squares, and ŷ is calculated from the fit. The selected polynomial, meeting predefined criteria, took the form of $y = ax_1+bx_2+cx_2(x_1-x_2)$. In this equation, y is the solid conversion rate, x_1 represents temperature in ^{o}C, and x_2 represents time in hour. The corresponding fitting parameters yielded an r^2 value exceeding 0.99 Notably, the coefficient a = 0.256 significantly below b = 6.907, emphasizing the pivotal influence of catalytic time in achieving high solid conversion rates. The c value is close to 0 (c = -0.016), indicating that the interaction of time and temperature has little effect on the solid conversion rate.be beneficial.

Supplementary Note 5. Thermodynamic feasibility

(1) Tandem of combustion-dehydrogenation

Fundamentally, the thermodynamic feasibility of long chain polyolefin reactions can be calculated and evaluated using Benson group increment theory (**Supplementary Table 4**). The thermodynamic contributions to a linear alkane C_nH_{2n+2} can be summarized as follow.

$$g_{alkanes} = (n-2)g_1 + 2g_2 \quad \text{(S1)}$$

A typical LDPE molecule (M_n = 1700, M_w = 4000) mainly contains 121 carbon atoms, so we calculate the entropy and enthalpy.

$$\Delta_f H_{alkanes} = -2584.40\ kJ \cdot mol^{-1} \quad \text{(S2)}$$

$$S_{m,alkanes} = 4.94\ kJ \cdot mol^{-1} \cdot K^{-1} \quad \text{(S3)}$$

We assume that aromatics products are mainly ortho-dominant, the thermodynamic contributions to aromatics C_nH_{2n-6} are shown as follows.

$$g_{aromatics} = (n-10)g_1 + 2g_2 + 4g_4 + 2g_5 + 2g_6 + g_7 \quad \text{(S4)}$$

$$\Delta_f H_{aromatics} = -2351.40\ kJ\ mol^{-1} \quad \text{(S5)}$$

$$S_{m,}\ aromatics = 4.81\ kJ\ mol^{-1}\ K^{-1} \quad \text{(S6)}$$

Aromatization of long chain PE are listed as follows.

$$C_nH_{2n+2} \rightarrow C_nH_{2n-6} + 4H_2 \quad \text{(S7)}$$

$$\Delta H_2 = \Delta_f H_{aromatics} + 4\Delta_f H_{hydrogen} - \Delta_f H_{alkanes} = 233.0\ kJ\ mol^{-1} \quad \text{(S8)}$$

$$\Delta S_2 = S_{m,}\ aromatics + 4S_{m,}\ hydrogen - S_{m,}\ alkanes = 392.8\ J\ mol^{-1}\ K^{-1} \quad \text{(S9)}$$

Combined with formulas S2 and S4-S9, the thermodynamic equation for aromatization reaction is ΔH_2 = 233.0 kJ mol^{-1}, ΔG_2 = 15.72 kJ mol^{-1} at 280 °C. These data indicate that the aromatization reaction at this temperature is thermodynamically unfavorable.

Similarly, when the reaction coupled with the H_2 combustion reaction (oxygen-mediated PE aromatization), the oxygen-mediated PE aromatization reaction switches thermodynamically favorable (ΔG_3=-853.0 kJ mol^{-1}).

$$C_nH_{2n+2} + 2O_2 \rightarrow C_nH_{2n-6} + 4H_2O(g) \quad \text{(S10)}$$

$$\Delta H_3 = \Delta H_2 + 4\Delta H_{com} = -734.2\ kJ\ mol^{-1} \quad \text{(S11)}$$

$$\Delta S_3 = 4S_{m,}\ H_2O(g) + S_{m,}\ aromatics - S_{m,}\ alkanes - 2S_{m,}\ O_2 = 214.8\ J\ mol^{-1}\ K^{-1} \text{(S12)}$$

(2) Thermodynamic feasibility analysis of the oxygen-mediated PE aromatization

Assuming x mol of O_2 is involved in the oxygen-mediated PE aromatization, the chemical equation in the oxygen-mediated PE aromatization is shown as follows.

$$C_nH_{2n+2} + xO_2 \rightarrow C_nH_{2n-6} + 2xH_2O(g) + (4-2x)H_2 \quad \text{(S13)}$$

$$\Delta H_4 = \Delta_f H_{aromatics} + 2x\Delta_f H_{H_2O} + (4-2x)\Delta_f H_{hydrogen} - \Delta_f H_{alkanes} - x\Delta_f H_{O_2} = 233 - 483.6x\ kJ\ mol^{-1} \quad \text{(S14)}$$

$$\Delta S_4 = S_{m,}\ aromatics + 2xS_{m,}\ H_2O(g) + (4-2x)S_{m,}\ hydrogen - S_{m,}\ alkanes - xS_{m,}\ O_2 = 394 - 87x\ J\ mol^{-1}\ K^{-1} \quad \text{(S15)}$$

At 280 ^oC, assuming $\Delta G = 0$, the value of x is calculated to be 0.0344. Based on our typical 1 g PE catalytic experiments, only 0.36 μL $H_2O(l)$ needs to be produced theoretically for us to achieve thermodynamic feasibility. In fact, the amount of water produced in our experiment far exceeded this value, further demonstrating that the oxygen-mediated PE aromatization system is thermodynamically feasible.

(3) Thermodynamic analysis of C-H activation in the oxygen-mediated PE aromatization

The thermodynamic calculation of C-H activation for long chain polyolefin reactions can be calculated using Benson group increment theory (**Supplementary Table 4**). The thermodynamic contributions to a linear alkane C_nH_{2n+2} can be summarized as follow.

$$g_{alkanes} = (n-2)g_1 + 2g_2 \quad \text{(S16)}$$

A typical LDPE molecule (M_n = 1700, M_w = 4000) mainly contains 121 carbon atoms, so we calculate the entropy and enthalpy.

$$\Delta_f H_{alkanes} = -2584.40\ kJ \cdot mol^{-1} \quad \text{(S17)}$$

$$S_{m,alkanes} = 4.94\ kJ \cdot mol^{-1} \cdot K^{-1} \quad \text{(S18)}$$

Similarly, the thermodynamic contributions to a linear alkene C_nH_{2n} can be summarized as follows.

$$g_{alkenes} = (n-5)g_1 + 2g_2 + g_3 \quad \text{(S19)}$$

$$\Delta_f H_{alkenes} = -2541.40\ kJ \cdot mol^{-1} \quad \text{(S20)}$$

$$S_{m,alkenes} = 4.86\ kJ \cdot mol^{-1} \cdot K^{-1} \quad \text{(S21)}$$

The C-H activation reaction ($C_nH_{2n+2} \longrightarrow C_nH_{2n} + H_2$) is the key step in the oxygen-mediated PE aromatization and listed as follows.

$$\Delta H_1 = \Delta_f H_{alkenes} + \Delta_f H_{hydrogen} - \Delta_f H_{alkanes} = 43.0\ kJ \cdot mol^{-1} \quad \text{(S22)}$$

$$\Delta S_1 = S_{m,alkenes} + S_{m,hydrogen} - S_{m,alkanes} = 57.0\ J \cdot mol^{-1} \cdot K^{-1} \quad \text{(S23)}$$

According to the contribution of the Benson group of long-chain n-alkanes and Geis' law, combined with the formulas of S16-S28, it can be deduced that the linear PE chain is converted into long-chain alkenes through the C-H activation pathway at 280 °C. The thermodynamic values are ΔH_1 = 43.0 kJ mol^{-1} and ΔG_1 = 11.47 kJ mol^{-1}, which suggest that C-H activation at this temperature is thermodynamically unfavorable. However, when the C-H activation reaction is coupled with the H_2 combustion reaction, the thermodynamic equation for C-H activation tandem hydrogen combustion is ΔH_2 = -198.82 kJ mol^{-1}, ΔG_2 = -246.44 kJ mol^{-1} at 280 ^oC. The above calculation indicates that the C-H activation in the oxygen-mediated PE aromatization is thermodynamic favorable.

$$H_2 + 0.5O_2 \rightarrow H_2O(g) \quad \text{(S24)}$$

$$\Delta H_{com} = -241.8\ kJ\ mol^{-1}\ K^{-1},\ \Delta S_{com} = -44\ J\ mol^{-1}\ K^{-1} \quad \text{(S25)}$$

$$C_nH_{2n+2} + 0.5O_2 \rightarrow C_nH_{2n} + H_2O(g) \quad \text{(S26)}$$

$$\Delta H_2 = \Delta H_1 + \Delta H_{com} = -198.8\ kJ\ mol^{-1} \quad \text{(S27)}$$

$$\Delta S_1 = S_{m,}\ H_2O(g) + S_{m,}\ alkenes - S_{m,}\ alkanes - 0.5S_{m,}\ O_2 = 86.1\ \text{J mol}^{-1}\ \text{K}^{-1} \quad \text{(S28)}$$

Supplementary Figures and Tables

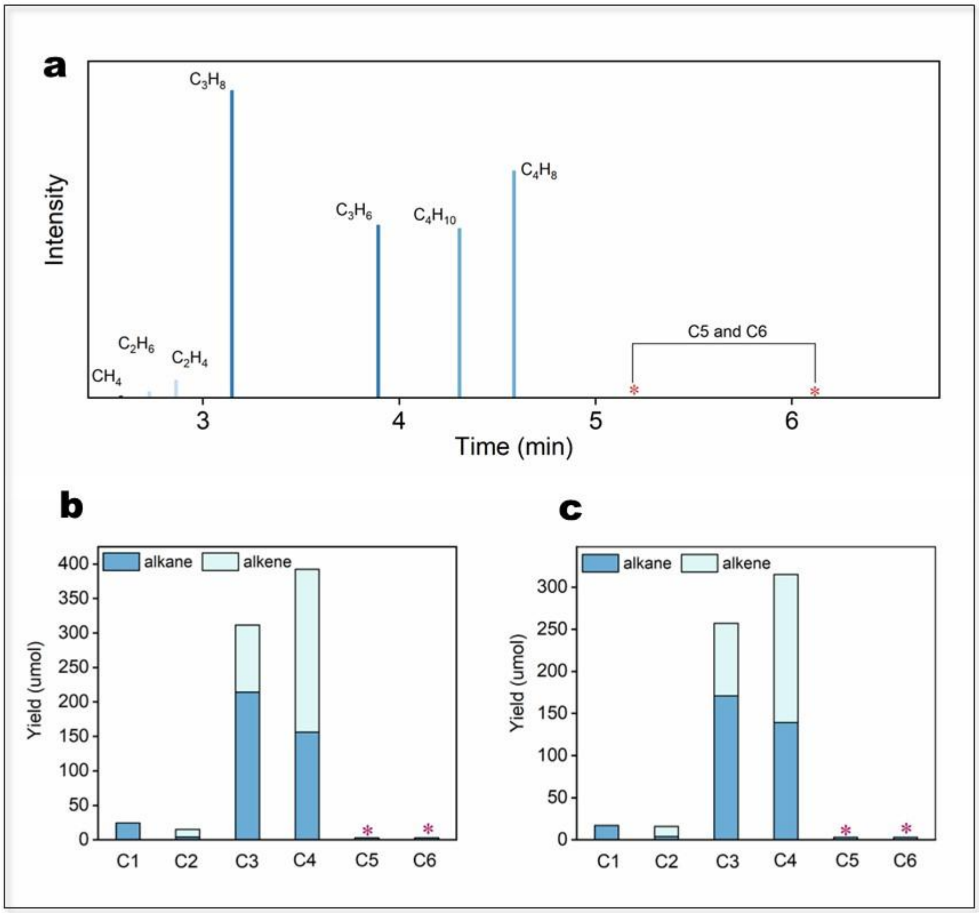

Supplementary Figure 1. (a) Typical GC spectrum of gas product distribution at 280 °C for 2 h over Ru/C+ZSM-5 catalyst. (b, c) The gas products distribution with the reaction in air (b) and N_2 (c). (Ru/C 50 mg, ZSM-5 200 mg, PE 2 g). * means that no obvious product is observed.

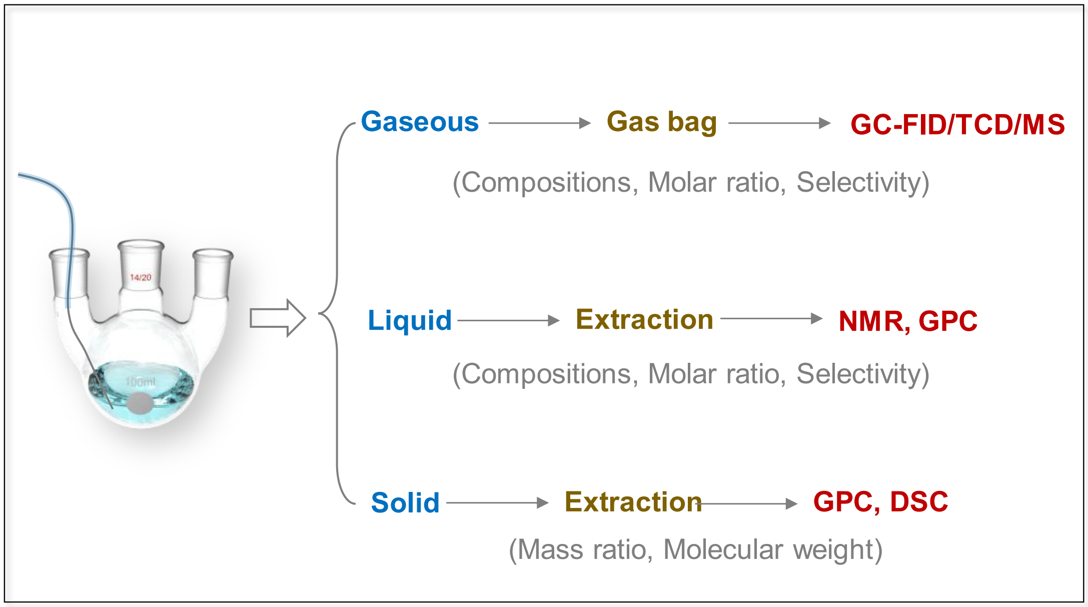

Supplementary Figure 2. Collection, qualitative and quantitative analysis of catalytic products.

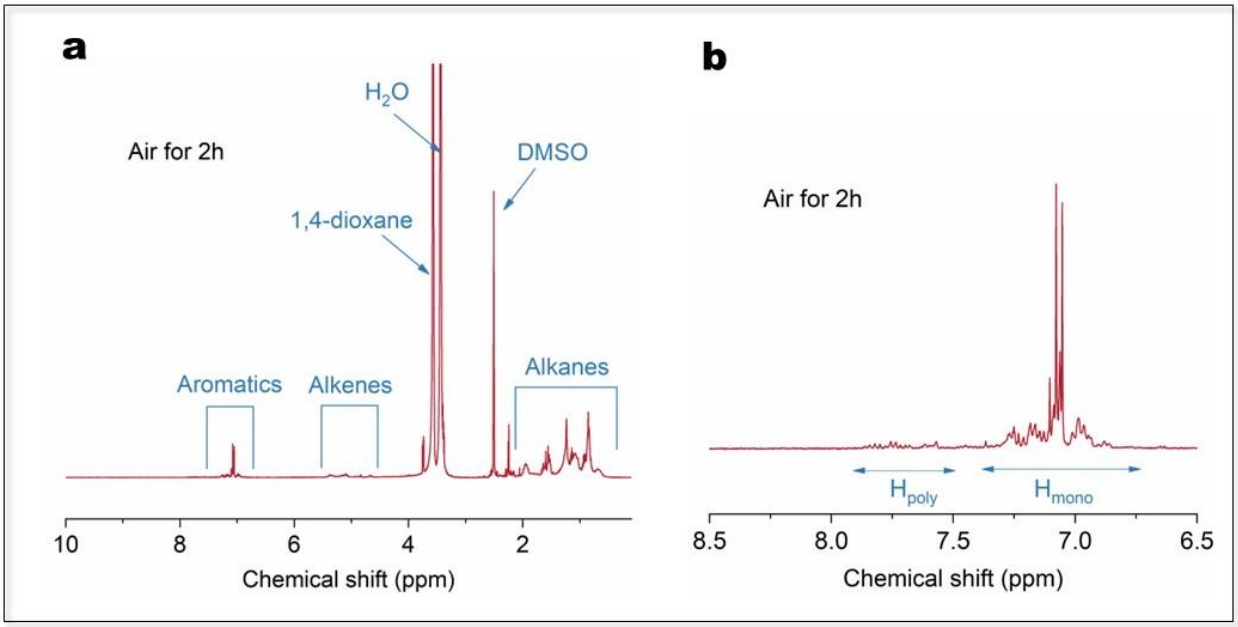

Supplementary Figure 3. ^{1}H NMR diagram of liquid products under air condition. Reaction conditions: T = 280 ^{o}C, t = 2 h, $m_{Ru/C}$ = 50 mg, m_{ZSM-5} = 200 mg, m_{PE} = 2 g.

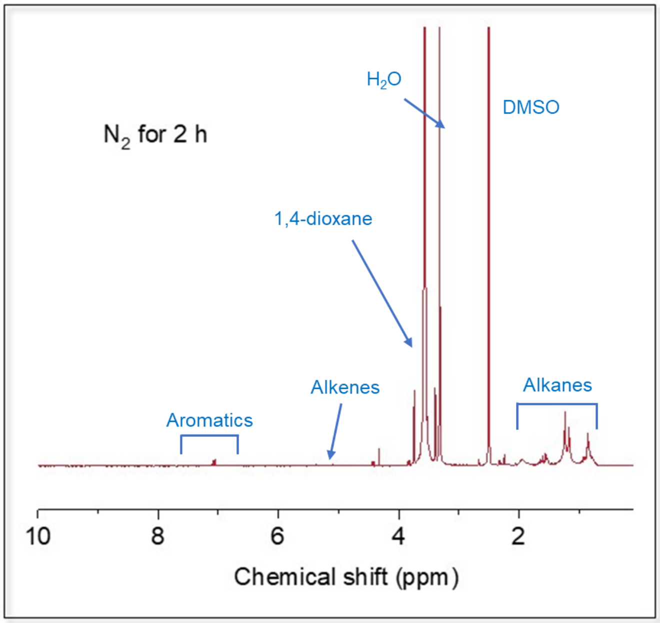

Supplementary Figure 4. ^{1}H NMR diagram of liquid products under N_2 condition. Reaction conditions: T = 280 ^oC, t = 2 h, $m_{Ru/C}$ = 50 mg, m_{ZSM-5} = 200 mg, m_{PE} = 2 g.

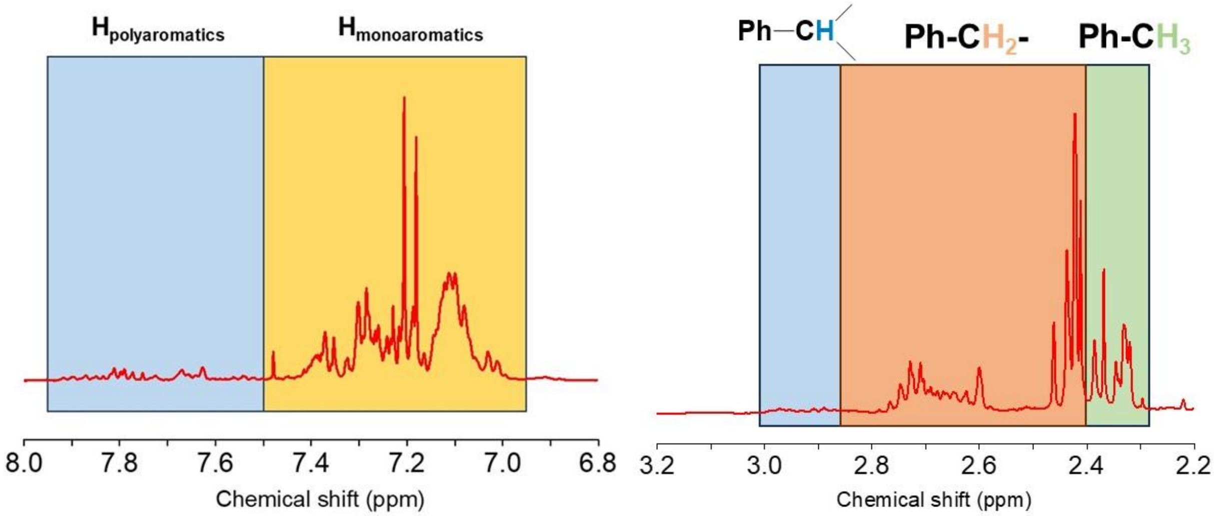

Supplementary Figure 5. ^{1}H NMR diagram of liquid products under air condition. Reaction conditions: T = 250 ^{o}C, t = 12 h, $m_{Ru/C}$ = 50 mg, m_{ZSM-5} = 200 mg, m_{PE} = 1 g, atmospheric pressure Air.

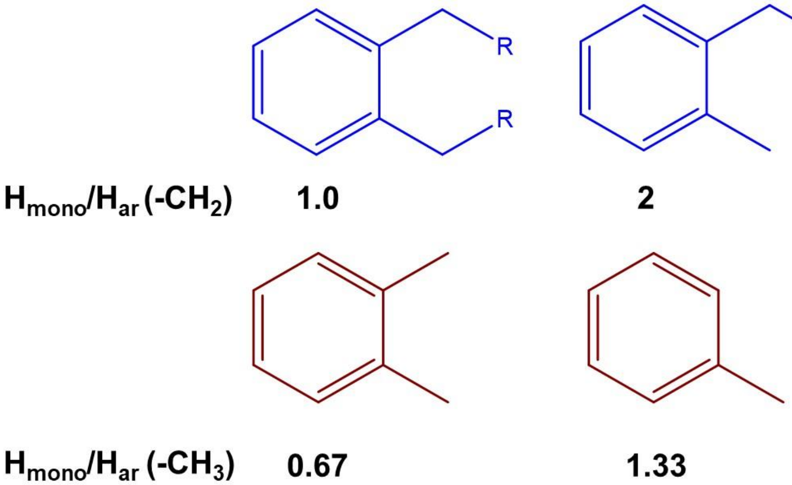

Supplementary Figure 6. The possible structures of mono-substituted and di-substituted alkylbenzene and their corresponding H_a/H_{ar} ratios, and the simulated molecular formulas of the products. Reaction conditions: T = 250 ^oC, t = 12 h, $m_{Ru/C}$ = 50 mg, m_{ZSM-5} = 200 mg, m_{PE} = 1 g, atmospheric pressure Air.

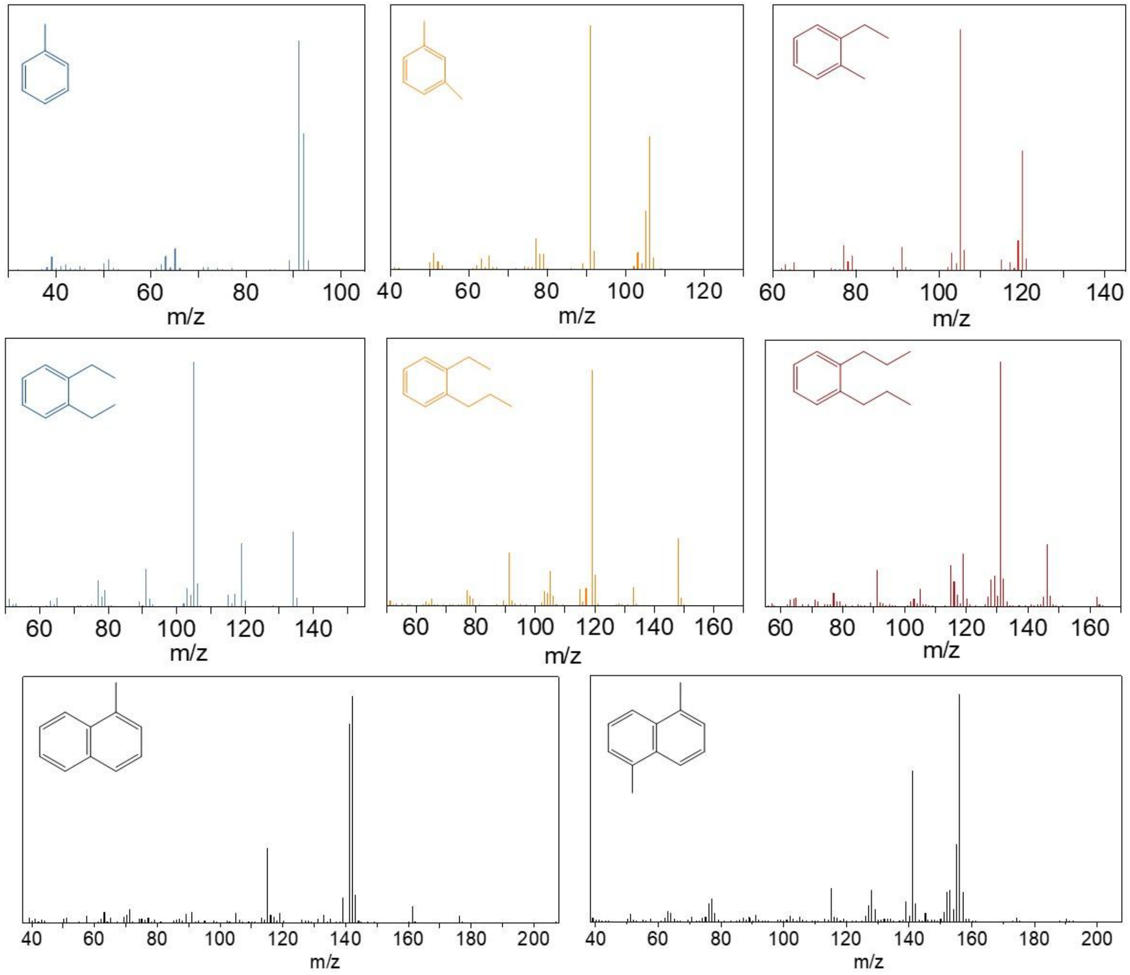

Supplementary Figure 7. MS spectra of representative products of the coupling reaction of PE and O_2. Reaction conditions: T = 250 ^oC, t = 12 h, $m_{Ru/C}$ = 50 mg, m_{ZSM-5} = 200 mg, m_{PE} = 1 g, atmospheric pressure O_2. Total liquid products were collected by THF.

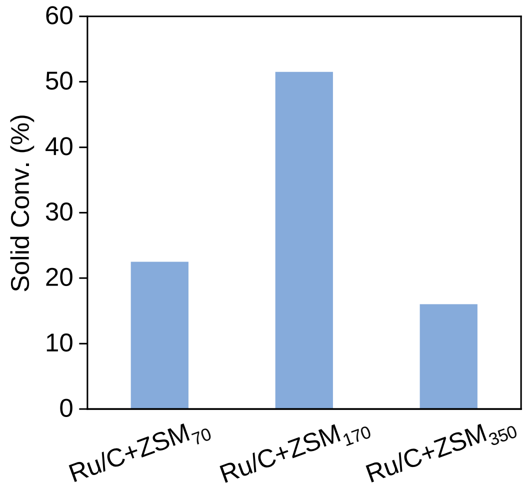

Supplementary Figure 8. Conversion rate of ZSM-5 zeolite with different Si/Al after physical mixing with commercial Ru/C. Reaction conditions: T = 280 °C, t = 2 h, $m_{Ru/C}$ = 50 mg, m_{ZSM-5} = 200 mg, m_{PE} = 2 g, atmospheric pressure O_2.

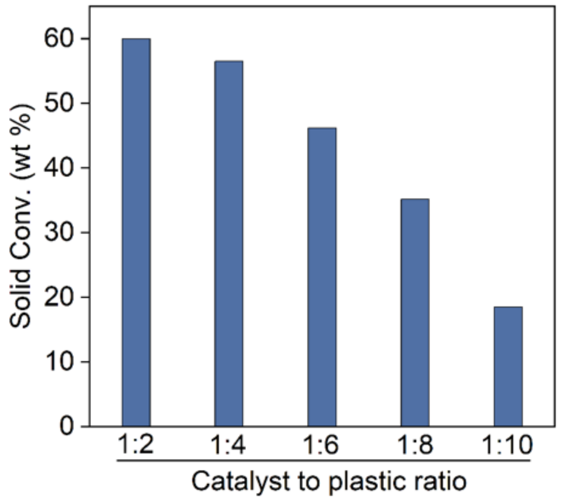

Supplementary Figure 9. Catalytic oxidation of PE over Ru/C and ZSM-5 (Si/Al ration=170) catalyst. Reaction conditions: T = 280 °C, t = 2 h, $m_{Ru/C}$ = 50 mg, m_{ZSM-5} = 200 mg.

We systematically investigated the effect of the catalyst-to-plastic ratio on the solid-phase conversion, and the results are shown in Supplementary Figure 9. The conversion rate strongly depends on the catalyst dosage, increasing from 18.5 wt% at a ratio of 1:10 to approximately 60.0 wt% at a ratio of 1:2. Higher conversion rates were observed at ratios of 1:2 and 1:4 (approximately 60 wt% and 56 wt%, respectively), while further reductions in catalyst dosage (≥1:6) led to a significant decrease in conversion. This phenomenon indicates that sufficient catalyst density is necessary to provide adequate metal and acidic sites for effective C-H bond activation, chain scission, and aromatization, while excessive catalyst dosage does not proportionally increase the conversion rate due to increased mass transfer and kinetic limitations. Considering both conversion efficiency and catalyst utilization, we selected a catalyst-to-plastic ratio of 1:4 as the optimal condition for subsequent experiments.

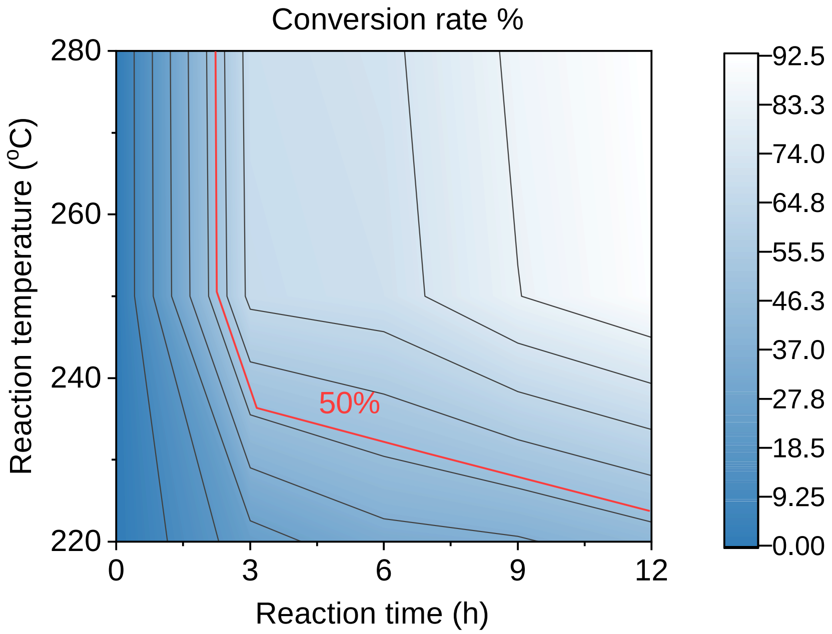

Supplementary Figure 10. Contour map of the effects of catalytic reaction time and temperature on solid conversion rate. Reaction conditions: mcatalyst = 250 mg, mPE = 1 g (Si/Al ratio of ZSM-5: 170).

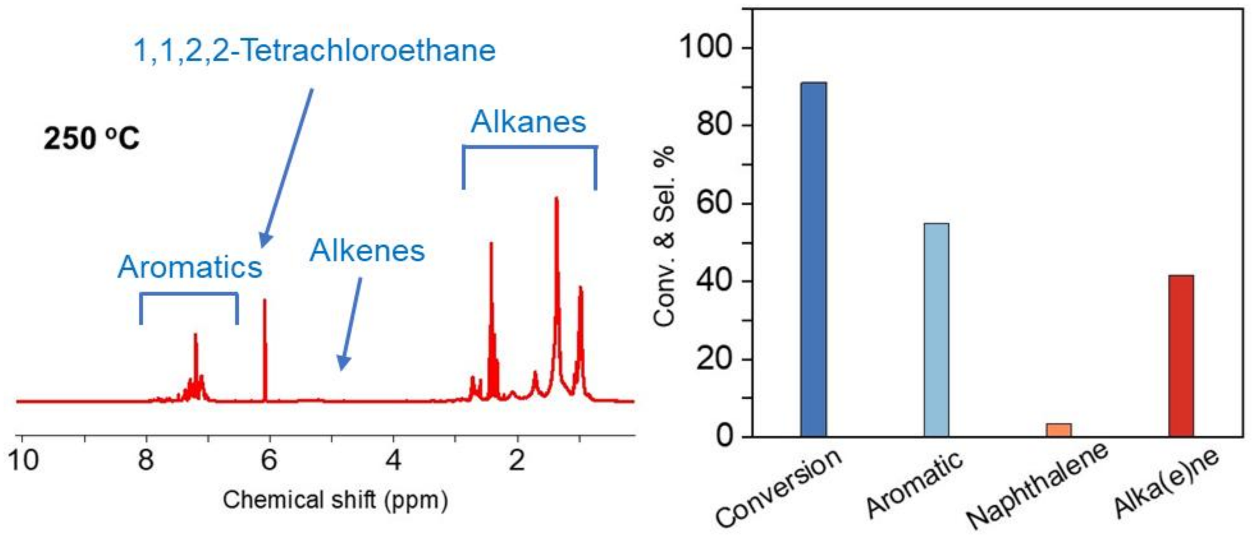

Supplementary Figure 11. ^{1}H NMR spectra (left) and product selectivity (right) of liquid products in oxygen-mediated PE aromatization reaction at low temperature. Reaction conditions: T = 250 °C, t = 12 h, $m_{Ru/C}$ = 50 mg, $m_{ZSM\text{-}5}$ = 200 mg, m_{PE} = 1 g, atmospheric pressure O_2.

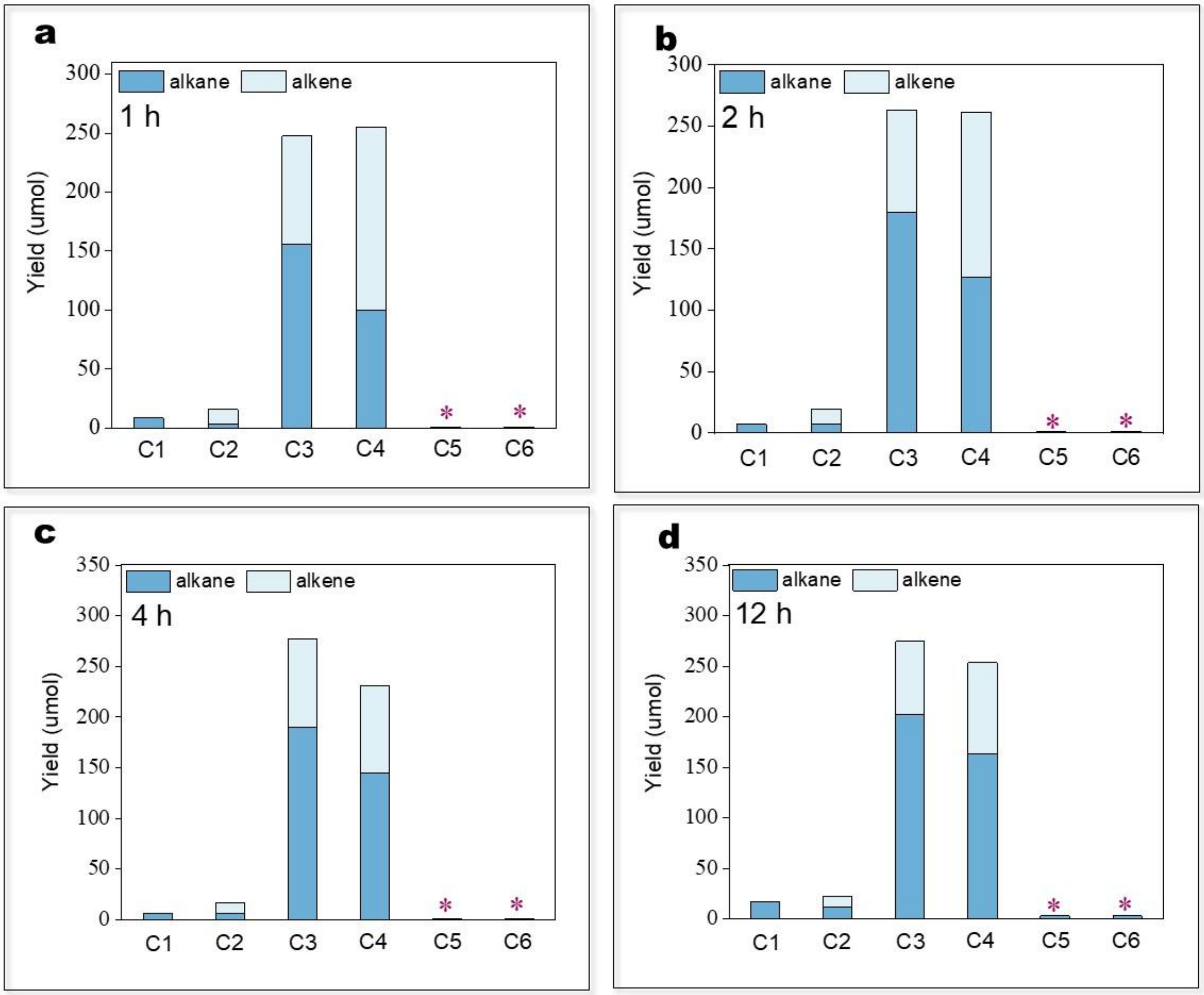

Supplementary Figure 12. (a, b, c, d) The gas products distributions. Reaction conditions: T = 280 °C, $m_{Ru/C}$ = 50 mg, $m_{ZSM\text{-}5}$ = 200 mg, m_{PE} = 1 g, atmospheric pressure O_2. * means that no obvious product is produced.

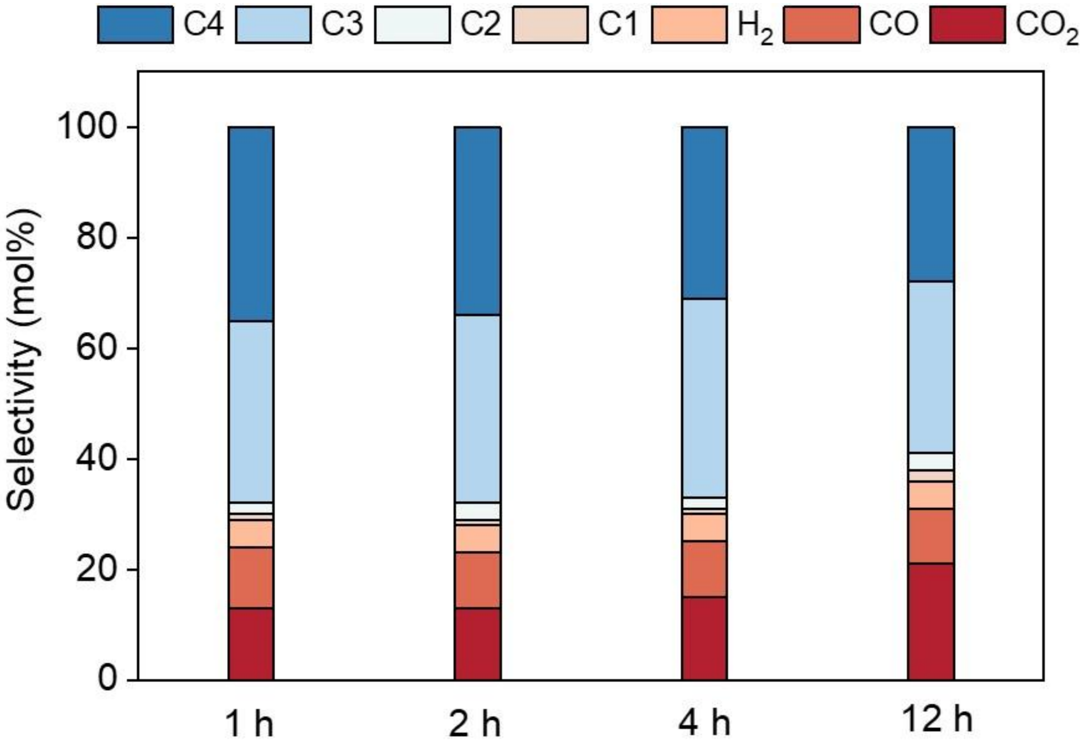

Supplementary Figure 13. The summarized gas products distributions. Reaction conditions: T = 280 ^{o}C, $m_{Ru/C}$ = 50 mg, m_{ZSM-5} = 200 mg, m_{PE} = 1 g, atmospheric pressure O_2.

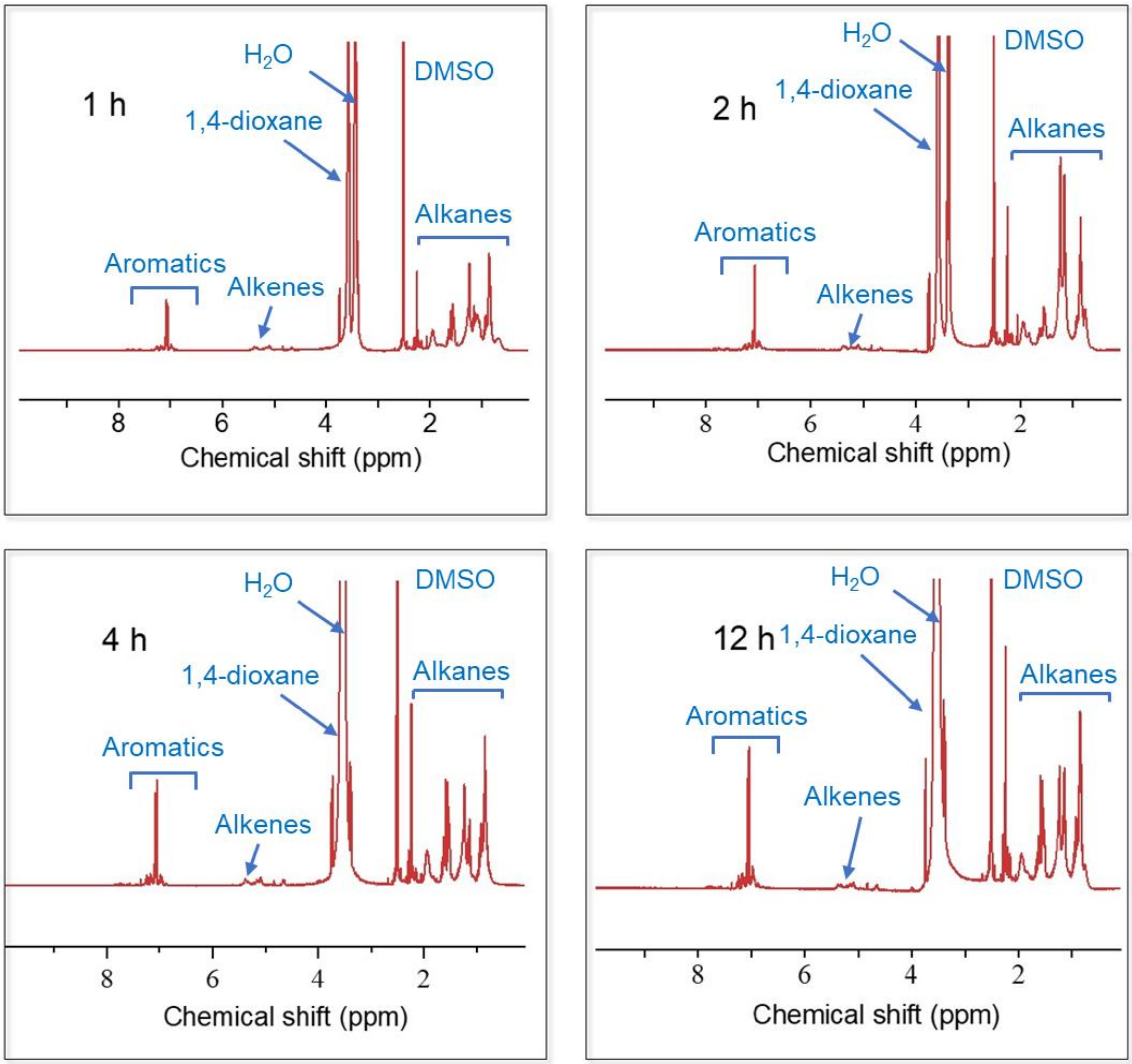

Supplementary Figure 14. ^{1}H NMR spectra of liquid products distributions. Reaction conditions: T = 280 ^{o}C, $m_{Ru/C}$ = 50 mg, m_{ZSM-5} = 200 mg, m_{PE} = 1 g, atmospheric pressure O_2.

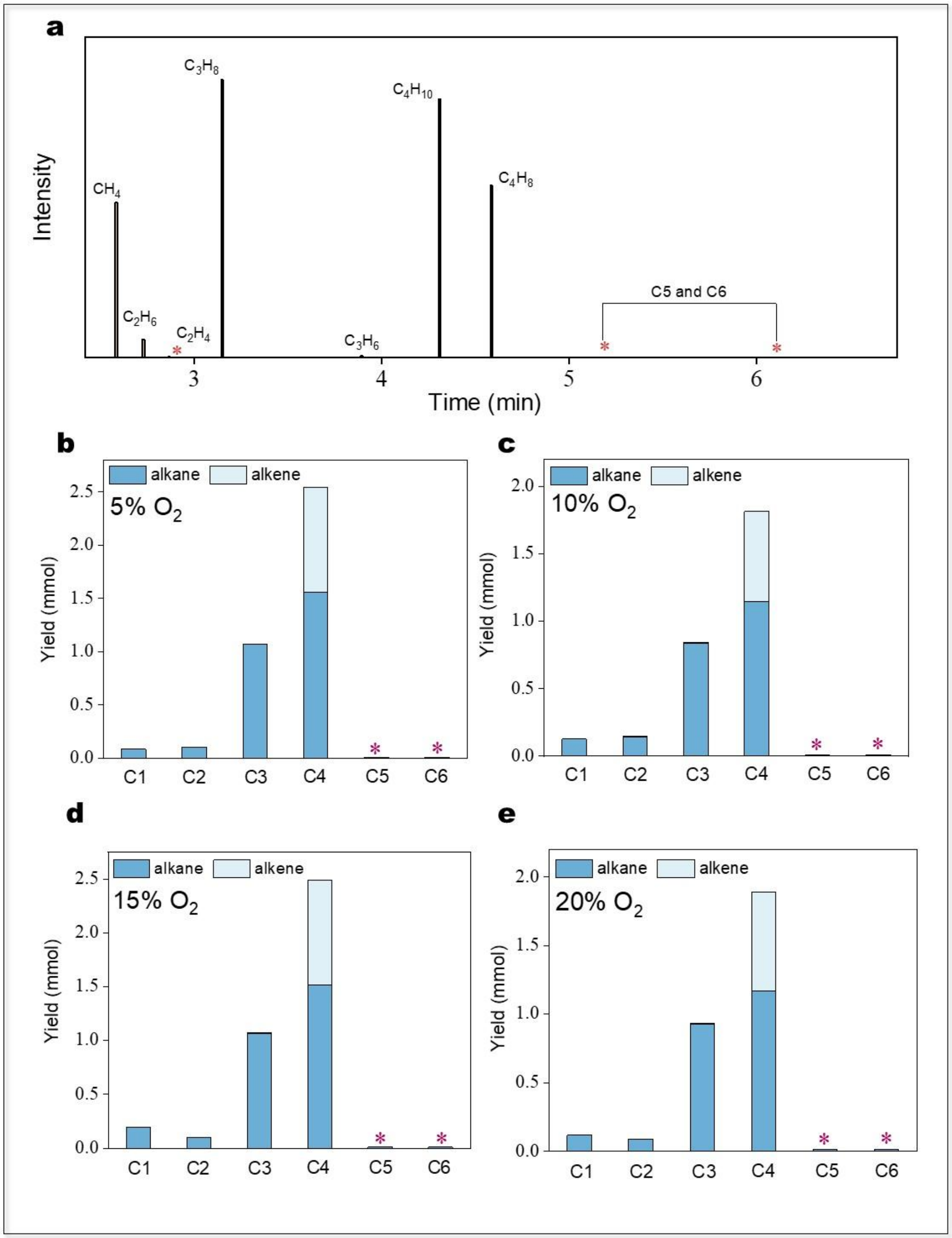

Supplementary Figure 15. (a) Typical GC spectrum of gas product distribution at 280 °C for 12 h over Ru/C+ZSM-5 catalyst with 20% O_2 concentration. (b, c, d, e) The gas products distributions. Reaction conditions: T = 280 °C, t = 12 h, $m_{Ru/C}$ = 50 mg, m_{ZSM-5} = 200 mg, m_{PE} = 1 g with different O_2 concentrations. * means that no obvious product is produced.

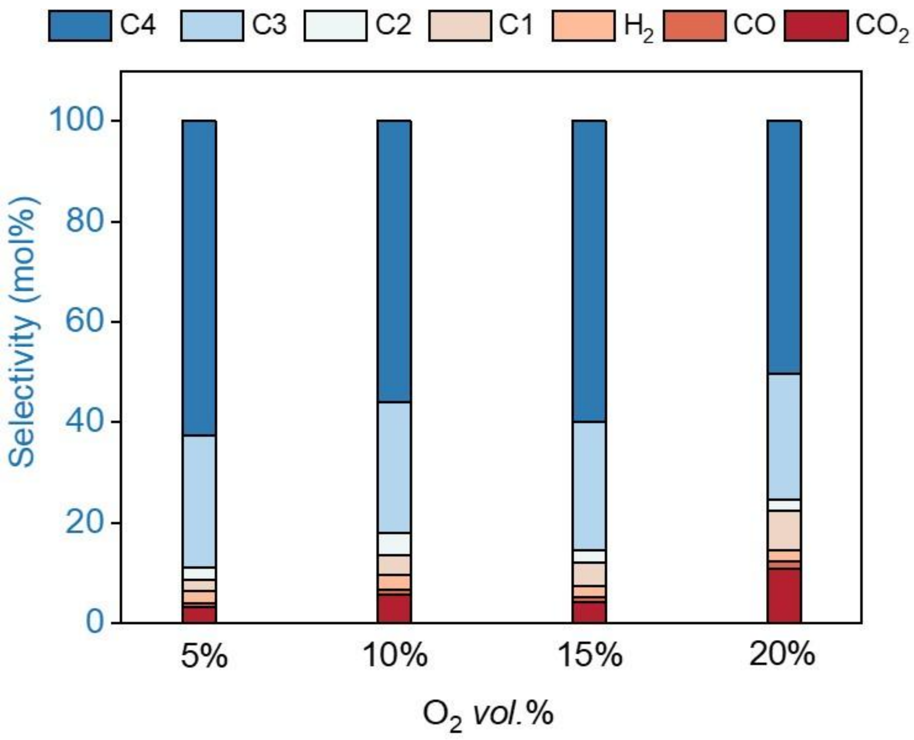

Supplementary Figure 16. The summarized gas products distributions. Reaction conditions: T = 280 ^{o}C, t = 12 h, $m_{Ru/C}$ = 50 mg, m_{ZSM-5} = 200 mg, m_{PE} = 1 g with different O_2 concentrations.

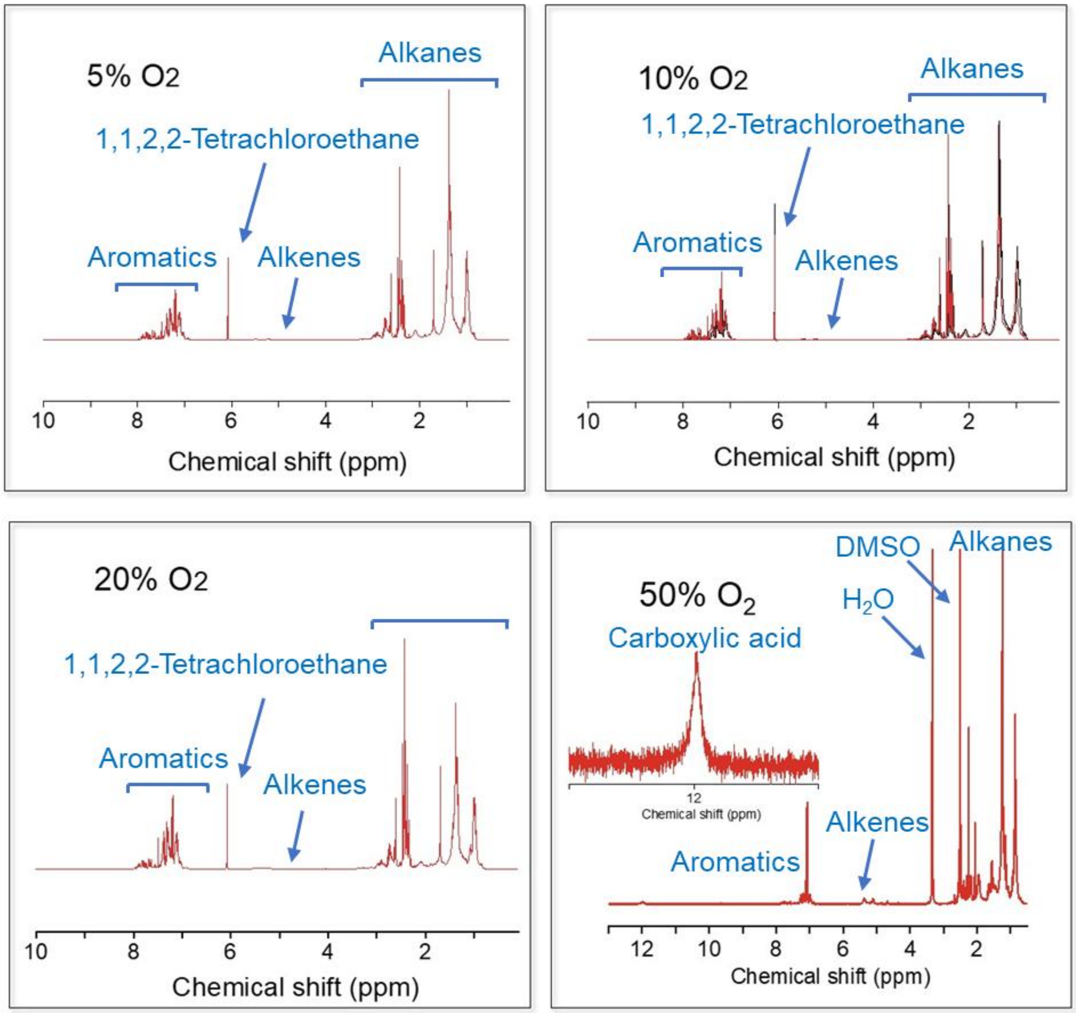

Supplementary Figure 17. ^{1}H NMR spectra of liquid products distributions. Reaction conditions: T = 280 °C, t = 12 h, $m_{Ru/C}$ = 50 mg, $m_{ZSM\text{-}5}$ = 200 mg, m_{PE} = 1 g with different O_2 concentrations.

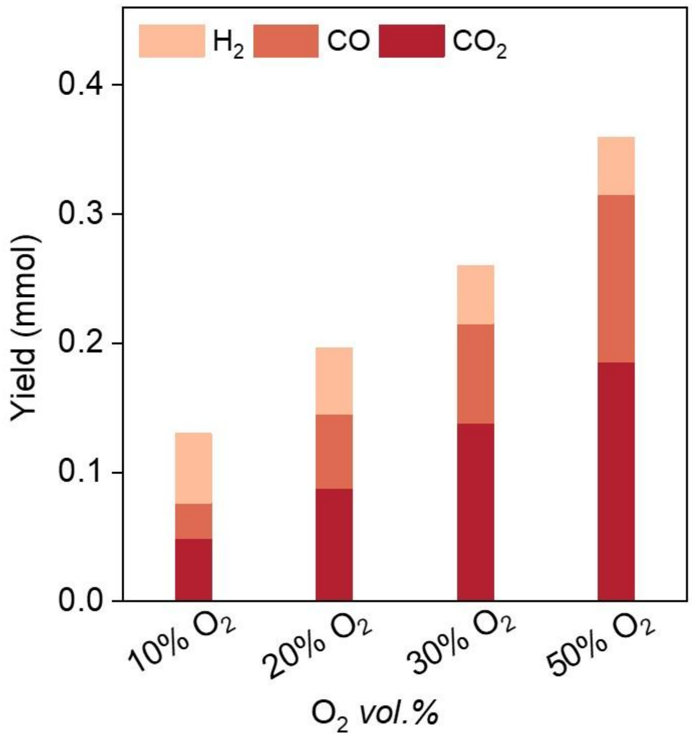

Supplementary Figure 18. The summarized gas products distributions. Reaction conditions: T = 280 ^{o}C, t = 12 h, $m_{Ru/C}$ = 50 mg, m_{ZSM-5} = 200 mg, m_{PE} = 1 g with different O_2 concentrations.

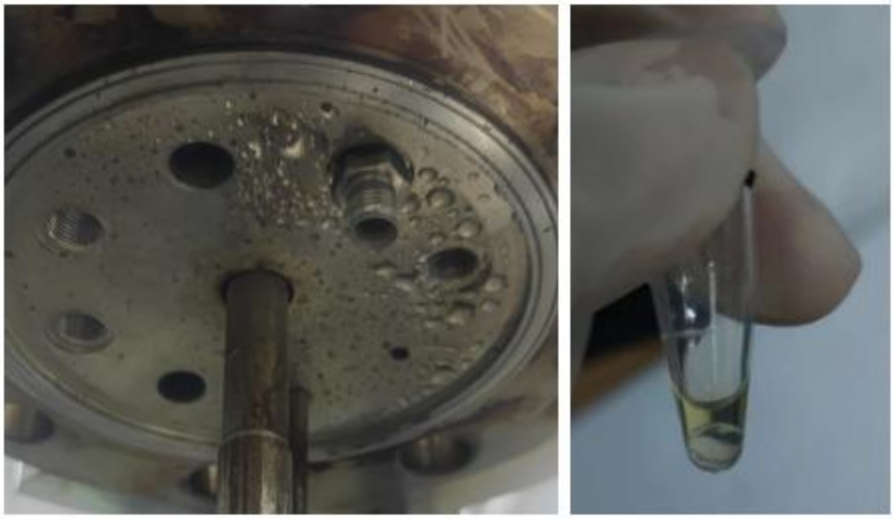

Supplementary Figure 19. Digital image showing the presence of water after the reaction.

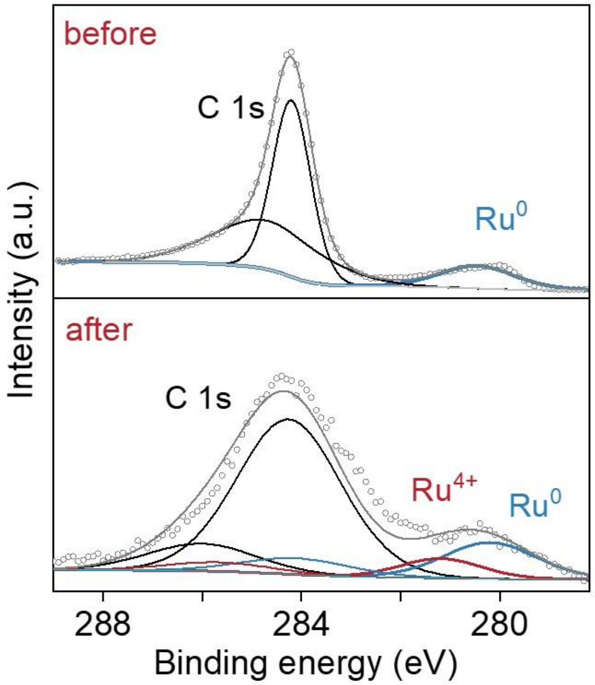

Supplementary Figure 20. High-resolution XPS spectra of Ru 3d for pristine Ru/C+ZSM-5 and spent catalyst after 280 ^{o}C for 12 h in air.

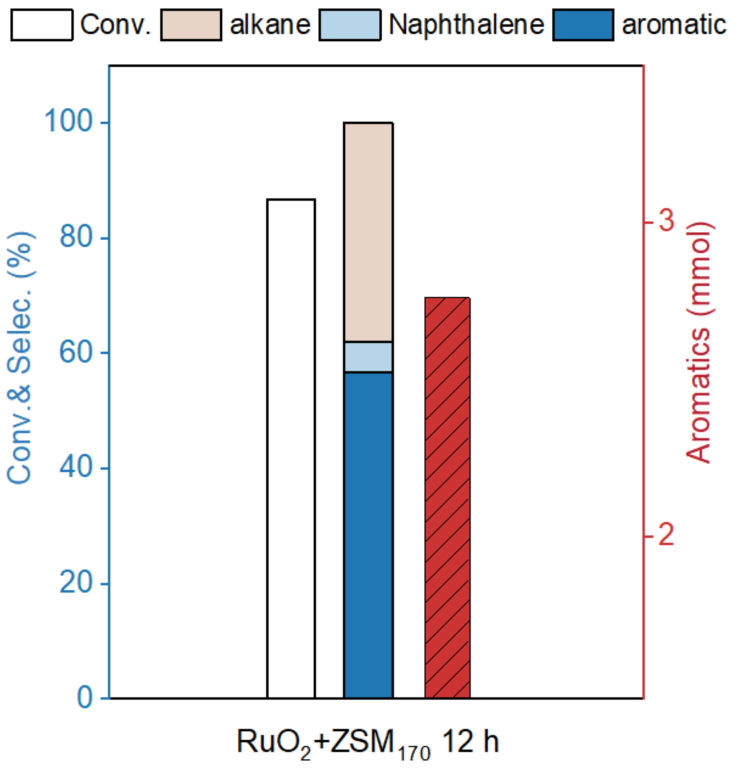

Supplementary Figure 21. The conversion rate and liquid product distribution over RuO_2+ZSM-5 at 280 ^{o}C for 12 h in air.

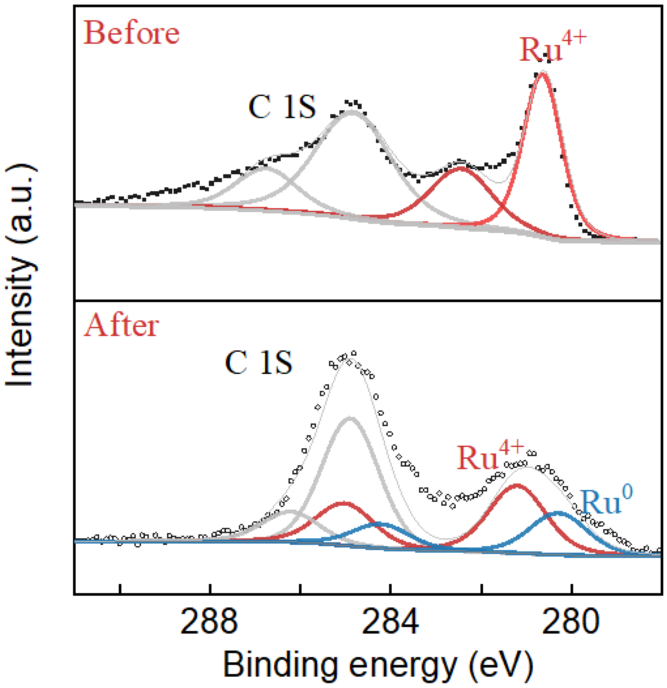

Supplementary Figure 22. High-resolution XPS spectra of Ru 3d for pristine RuO_2+ZSM-5 and spent catalyst after 280 °C for 12 h in air.

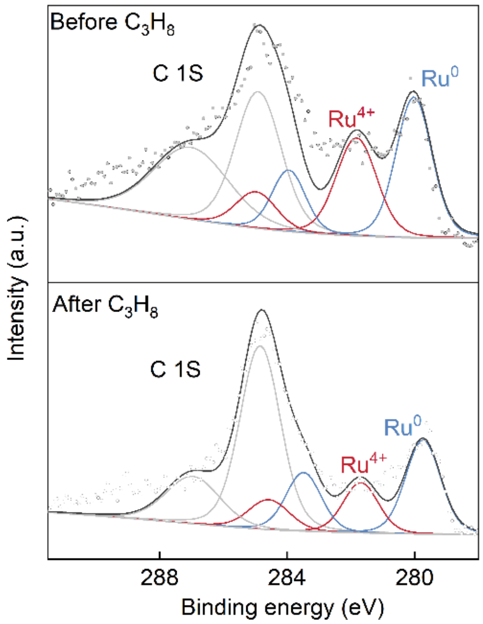

Supplementary Figure 23. High-resolution XPS spectra of Ru 3d for Ru/SiO_2 before and after propane treatment

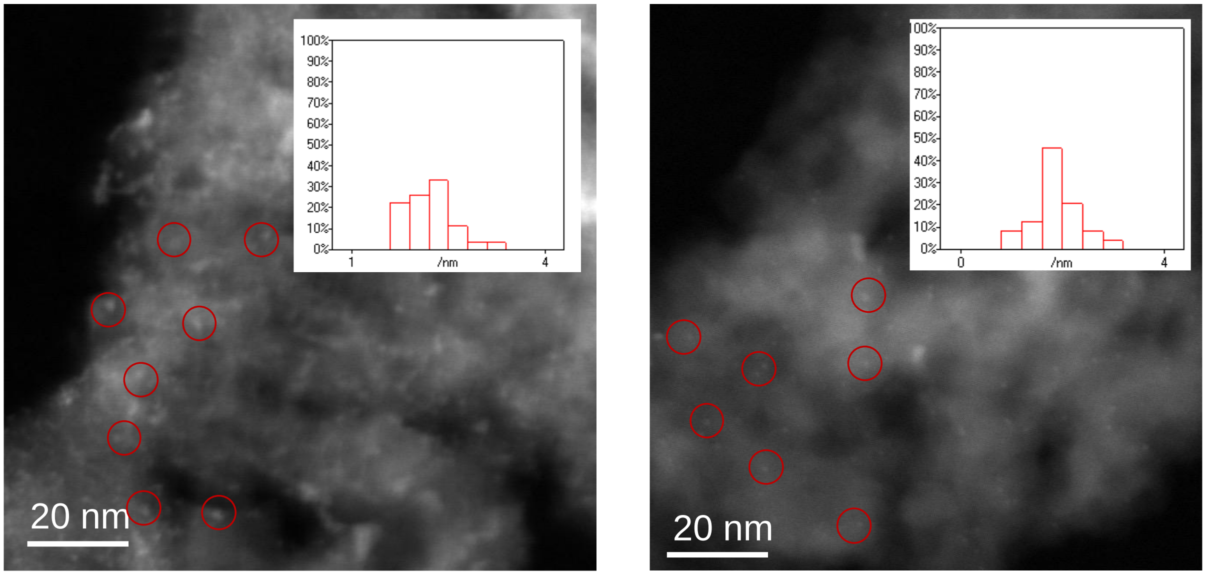

Supplementary Figure 24. TEM images and size distribution of Ru for pristine (left) and spent (right) catalysts after 280 ^{o}C for 12 h in air.

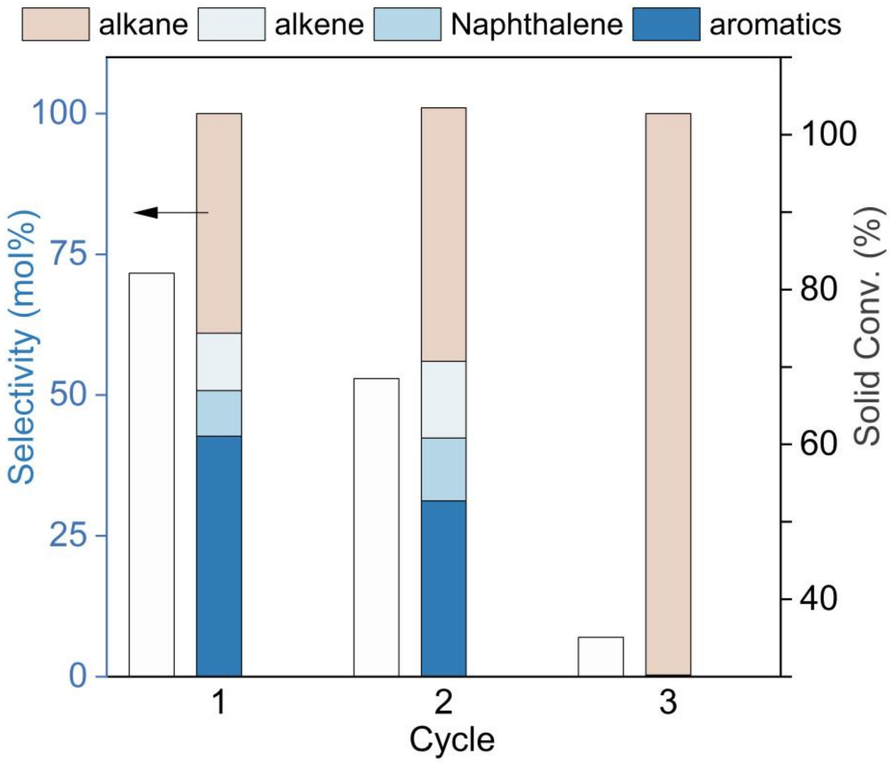

Supplementary Figure 25. The variation of the conversion and liquid product distribution of the Oxygen-Mediated PE Aromatization in three cycles under nitrogen atmosphere. Reaction conditions: T = 280 ^{o}C, t = 12 h, $m_{Ru/C}$ = 50 mg, $m_{ZSM\text{-}5}$ = 200 mg, m_{PE} = 1 g with Nitrogen at atmospheric pressure.

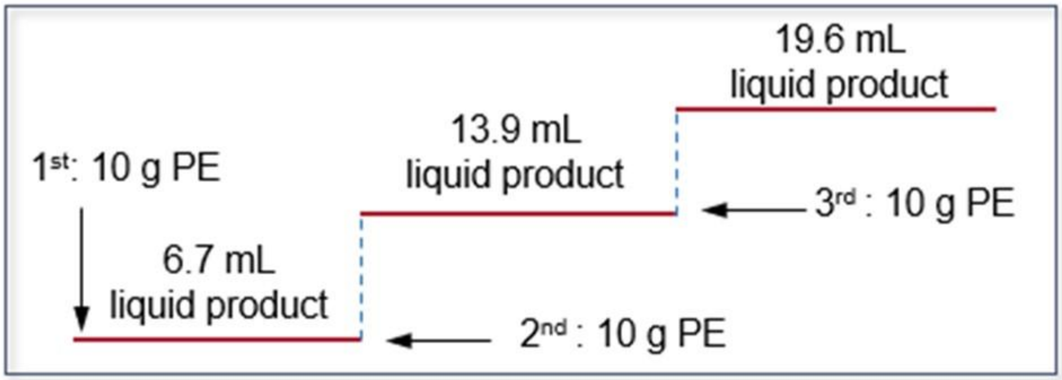

Supplementary Figure 26. A batch catalytic reaction. 10 g of LDPE was added to the three-necked flask at 2-hour intervals for each cycle, without any intervening catalyst treatment or regeneration. Reaction conditions: T = 280 ºC, t = 2 h, $m_{Ru/C}$ = 0.5 g, m_{ZSM-5} = 2 g, m_{PE} = 10 g under air atmosphere.

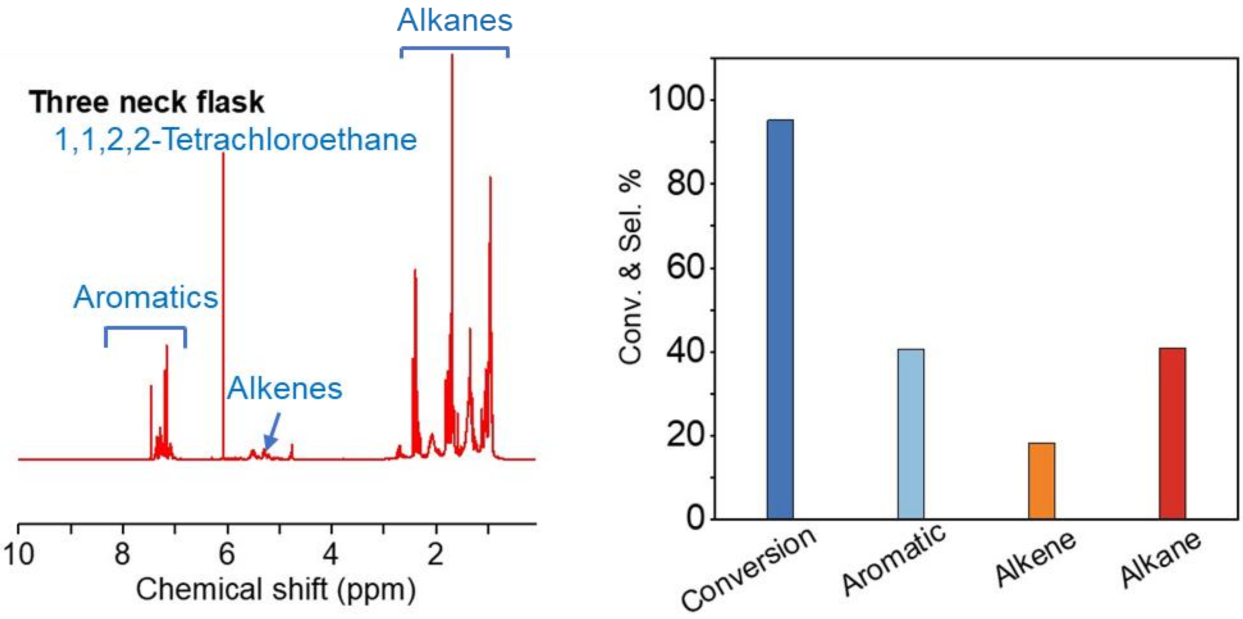

Supplementary Figure 27. ^{1}H NMR spectra (left) and product selectivity of liquid products (right) in catalytic reactions. Reaction conditions: T = 280 ^oC, t = 2 h, $m_{Ru/C}$ = 0.5 g, m_{ZSM-5} = 2 g, m_{PE} = 10 g under air atmosphere. (10 g of LDPE was added to the three-necked flask at 2-hour intervals for each of three cycles)

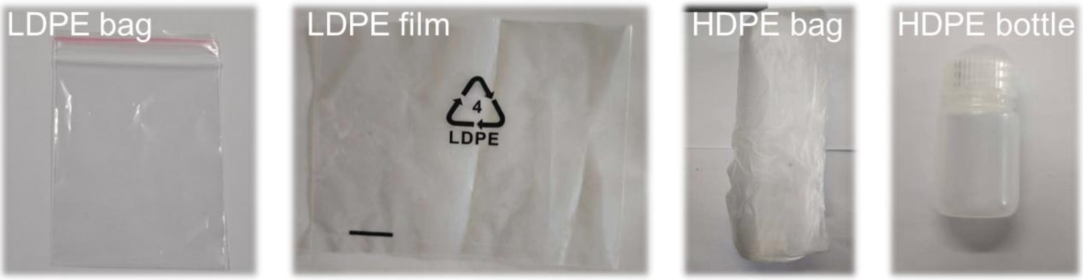

Supplementary Figure 28. Digital photograph of real polyethylene plastic.

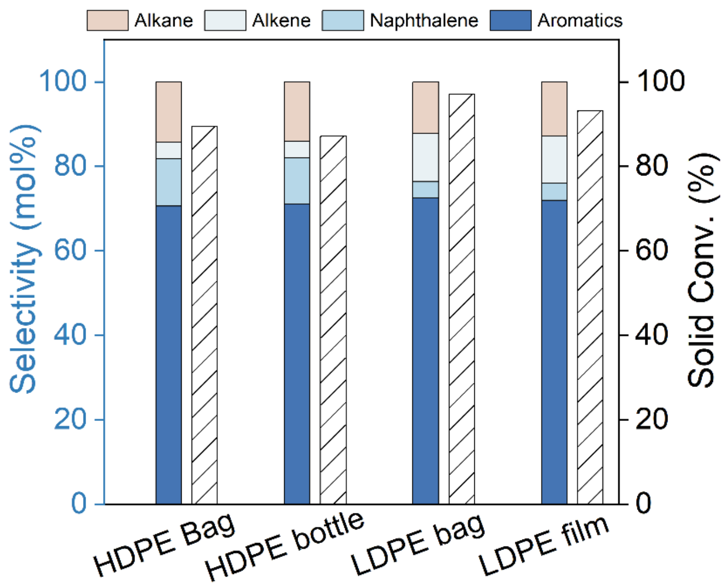

Supplementary Figure 29. Selectivity of liquid-phase products and solid conversion in oxygen-mediated aromatization of real polyethylene plastics at low temperatures. Reaction conditions: T = 280 °C, t = 12 h, $m_{Ru/C}$ = 50 mg, m_{ZSM-5} = 200 mg, m_{PE} = 1 g, atmospheric oxygen.

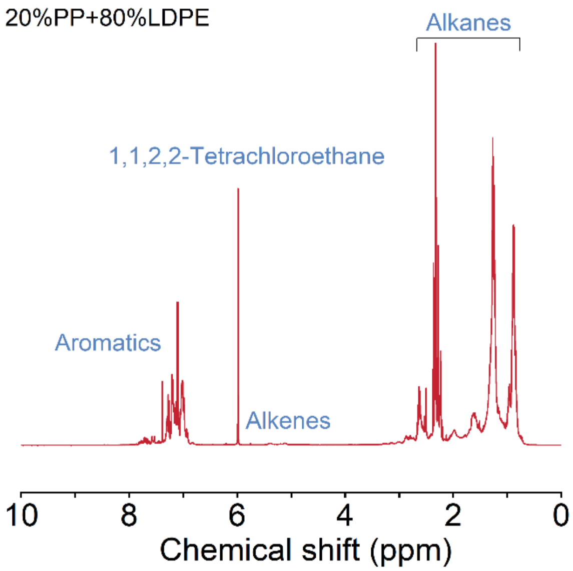

Supplementary Figure 30. ^{1}H-NMR diagram of high-density polyethylene aromatization.

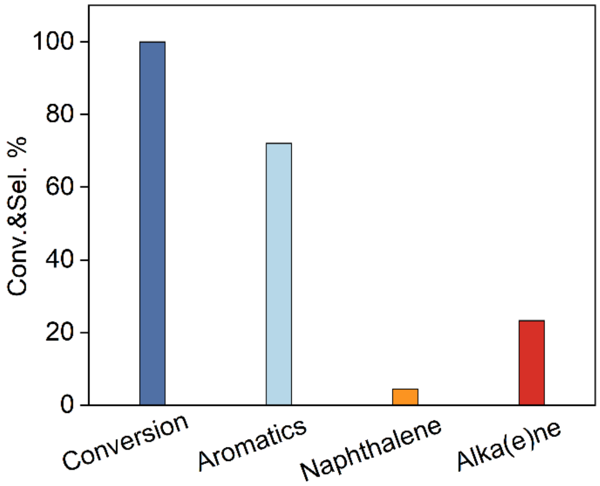

Supplementary Figure 31. Selectivity of liquid-phase products in oxygen-mediated aromatization reaction of PP (20%) and LDPE (80%) blends at low temperature. Reaction conditions: T = 280 °C, t = 10 h, $m_{Ru/C}$ = 50 mg, m_{ZSM-5} = 200 mg, m_{PE+PP} = 1 g, atmospheric oxygen.

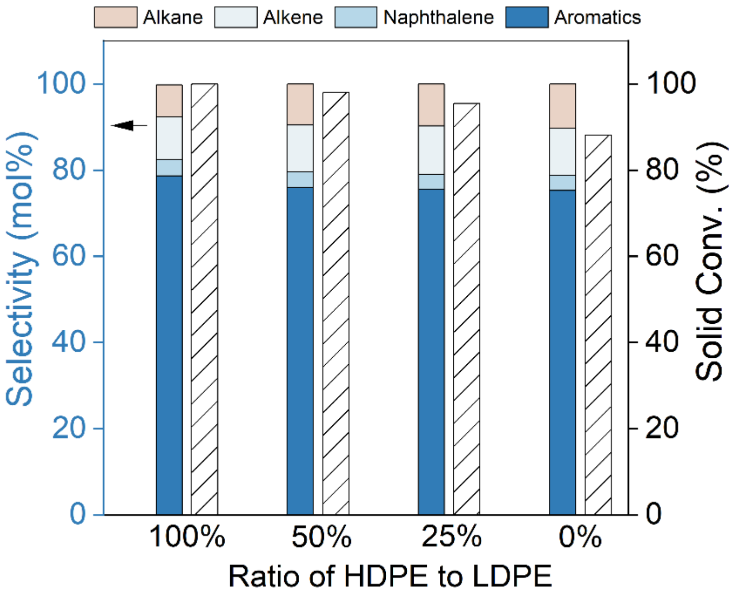

Supplementary Figure 32. Selectivity of liquid-phase products in oxygen-mediated aromatization reaction of LDPE and HDPE blends at low temperatures. Reaction conditions: T = 280 °C, t = 10 h, $m_{Ru/C}$ = 50 mg, m_{ZSM-5} = 200 mg, m_{HDPE} = 1 g, atmospheric oxygen.

We systematically investigated the aromatization performance of LDPE/HDPE mixtures with different mass ratios (Supplementary Figure 32). As the feedstock composition varied from pure HDPE to mixtures containing 50% and 25% HDPE, and finally to pure LDPE, the aromatic selectivity gradually decreased from 78.7 mol% to 76.0 mol%, 75.6 mol%, and 75.3 mol%, respectively, while remaining above 75 mol% in all cases. Notably, the presence of HDPE also shortened the reaction time required for complete conversion from 12 h to 10 h. This behavior is attributed to the lower branching degree of HDPE, which facilitates chain stacking, cyclization, and aromatization. These results demonstrate the strong tolerance of the catalytic system toward mixed PE feedstocks and its robustness under realistic plastic compositions.

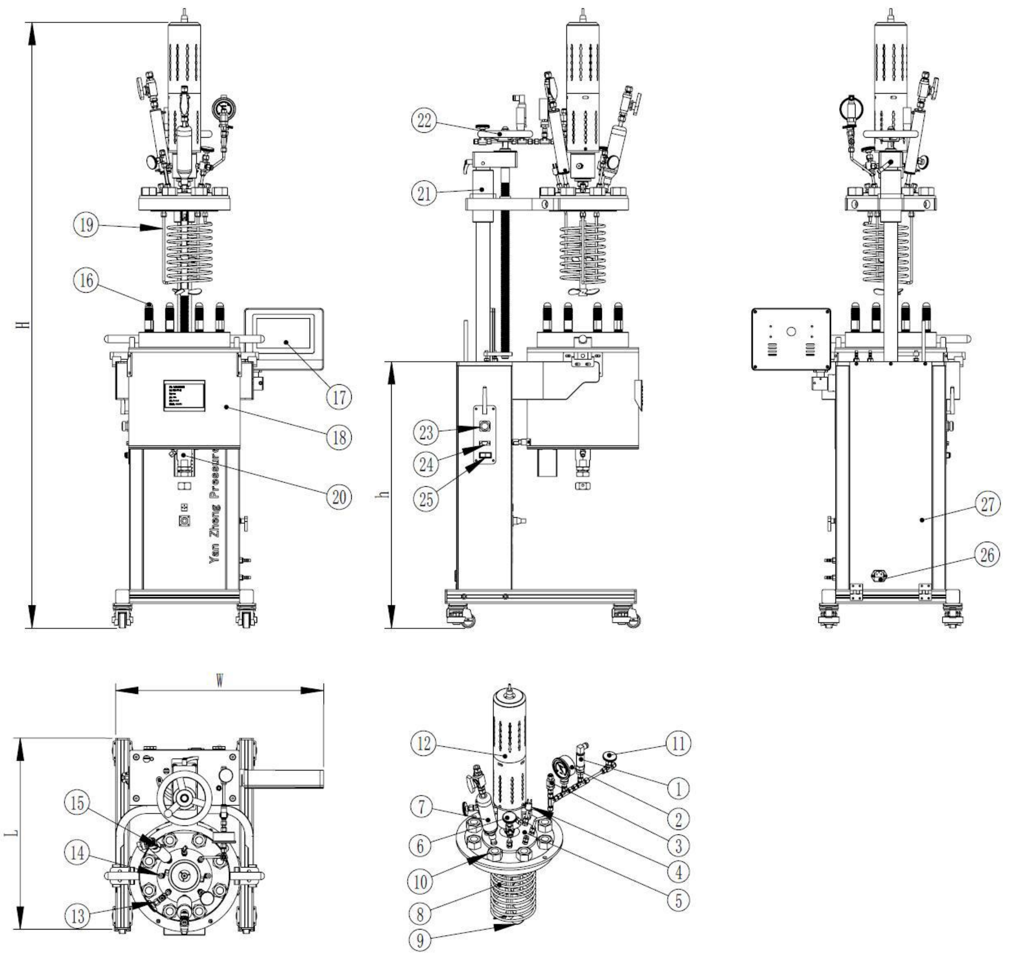

Supplementary Figure 33. Schematic illustration of the 2 L reactor.

No.	Component	No.	Component
**1**	Pressure sensor	**2**	pressure gauge
**3**	safety valve	**4**	Temperature sensor
**5**	Reactor lid	**6**	liquid phase valve
**7**	constant pressure feeding window	**8**	cooling coil
**9**	stirrer	**10**	nut
**11**	exhaust valve	**12**	magnetic coupling stirring
**13**	air inlet valve	**14**	cooling water inlet and outlet
**15**	condensation return pipe	**16**	bolts
**17**	operation panel	**18**	heating furnace
**19**	cooling coil	**20**	bottom valve
**21**	lifting module	**22**	handwheel
**23**	network port	**24**	RS485 communication port
**25**	switch	**26**	power in

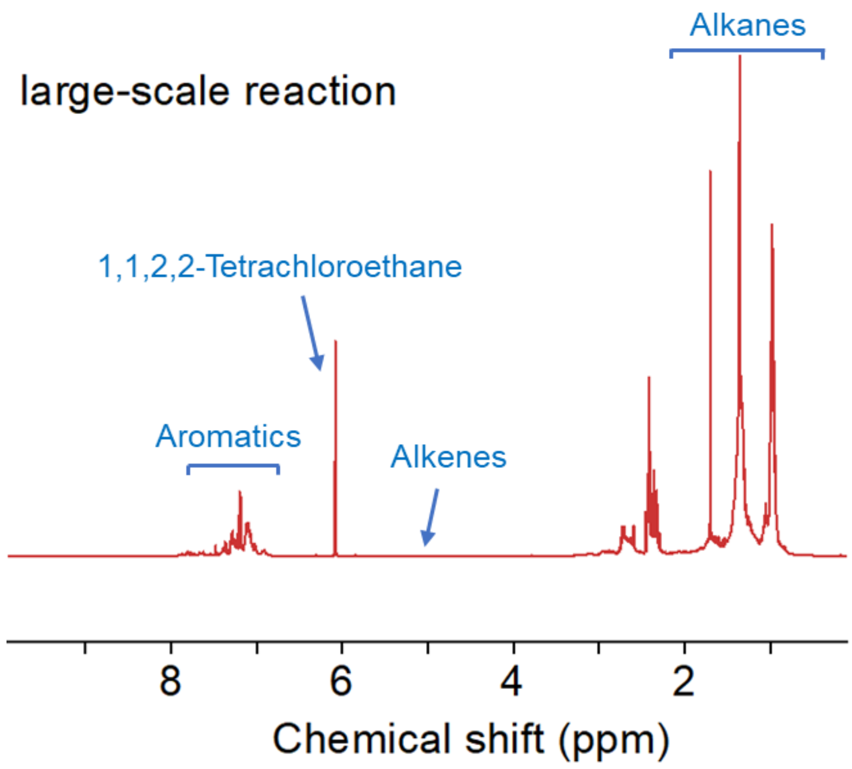

Supplementary Figure 34. ^{1}H NMR spectrum of liquid product in large-scale reaction. Reaction conditions: T = 280 ^{o}C, t = 12 h, $m_{catalyst}$ = 25 g, m_{PE} = 100 g (Si/Al ratio of ZSM-5: 170).

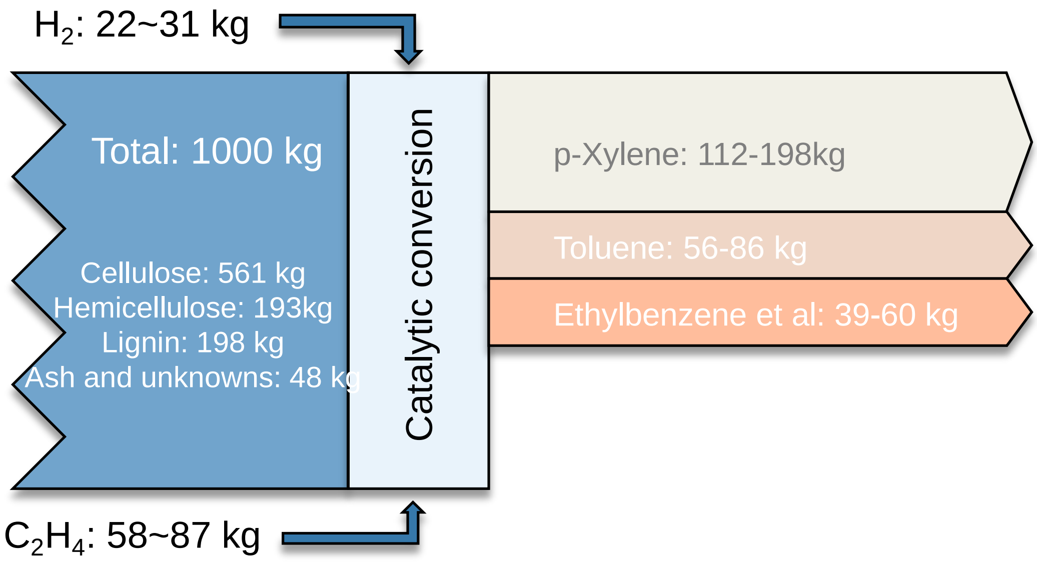

Supplementary Figure 35. Mass balance of biomass into aromatics (assuming the conversion of 1 ton feedstocks, cited from *Angew. Chem. Int. Ed.*,[8] **2021**, *133*, 5587-5595).

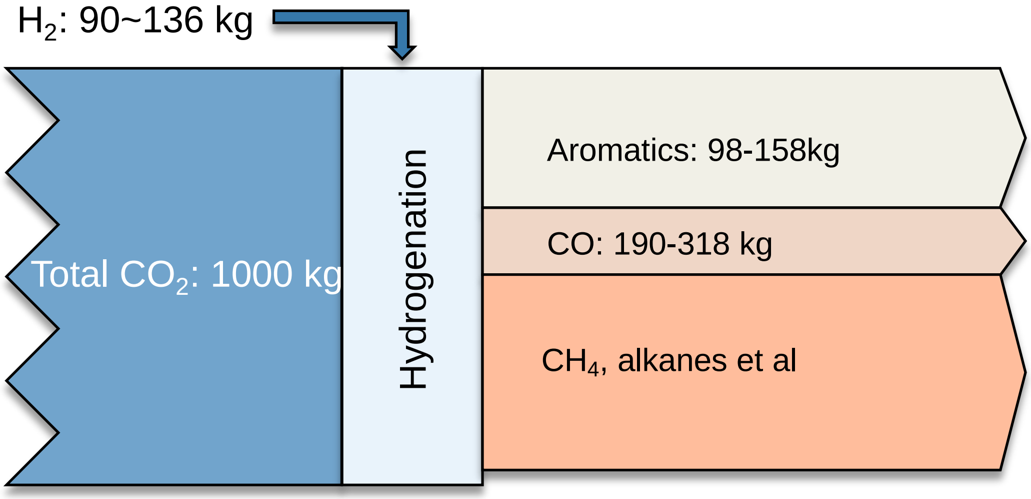

Supplementary Figure 36. Mass balance of CO_2 into aromatics (assuming the conversion of 1 ton feedstocks,[8] cited from *Angew. Chem. Int. Ed.*, **2021**, *133*, 5587-5595).

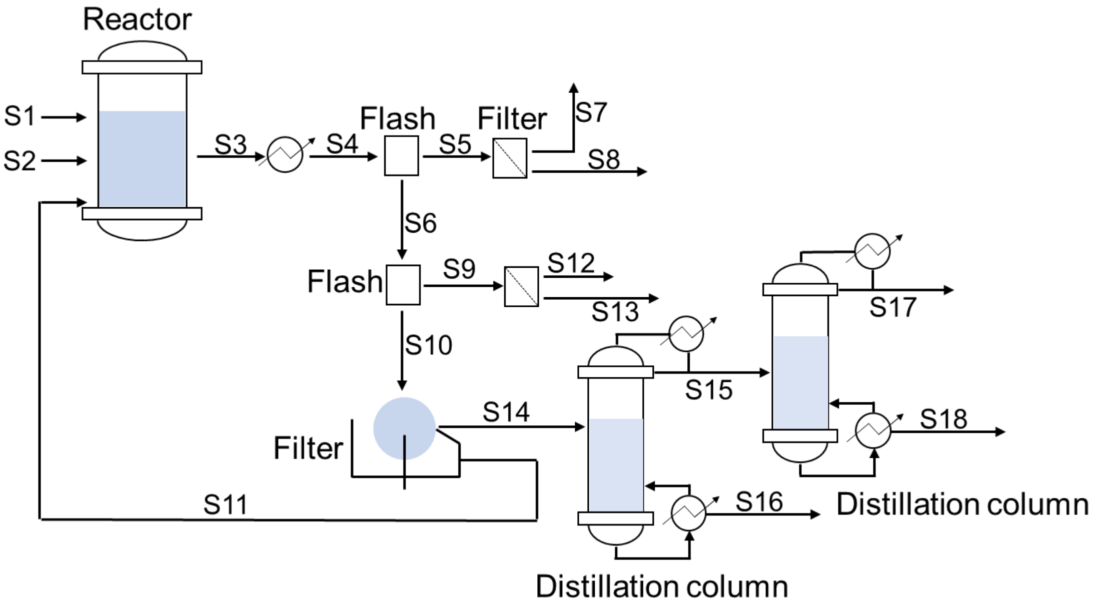

Supplementary Figure 37. Simplified process flow diagram of a typical thermo-catalytic PE oxidation process.

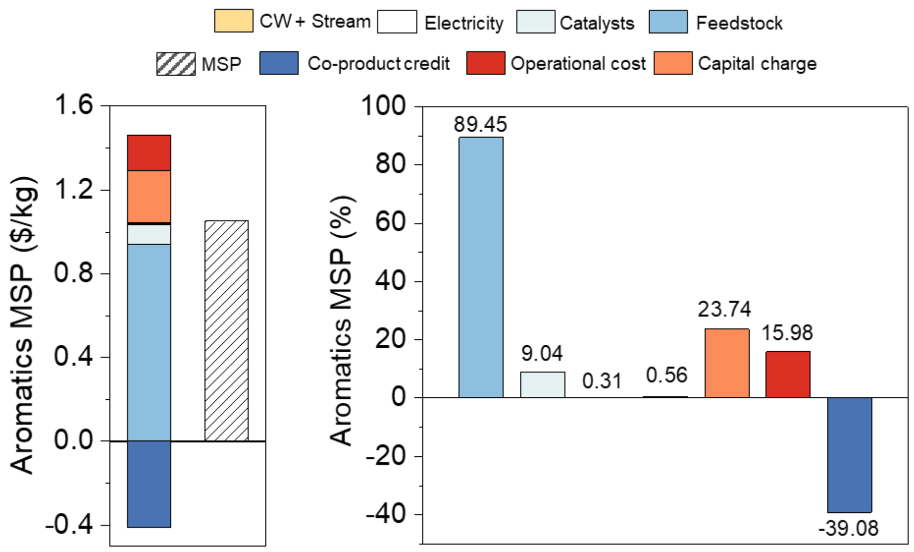

Supplementary Figure 38. Cost breakdown of the aromatics MSP in the base case process and MSP with percentage contribution of each factor.

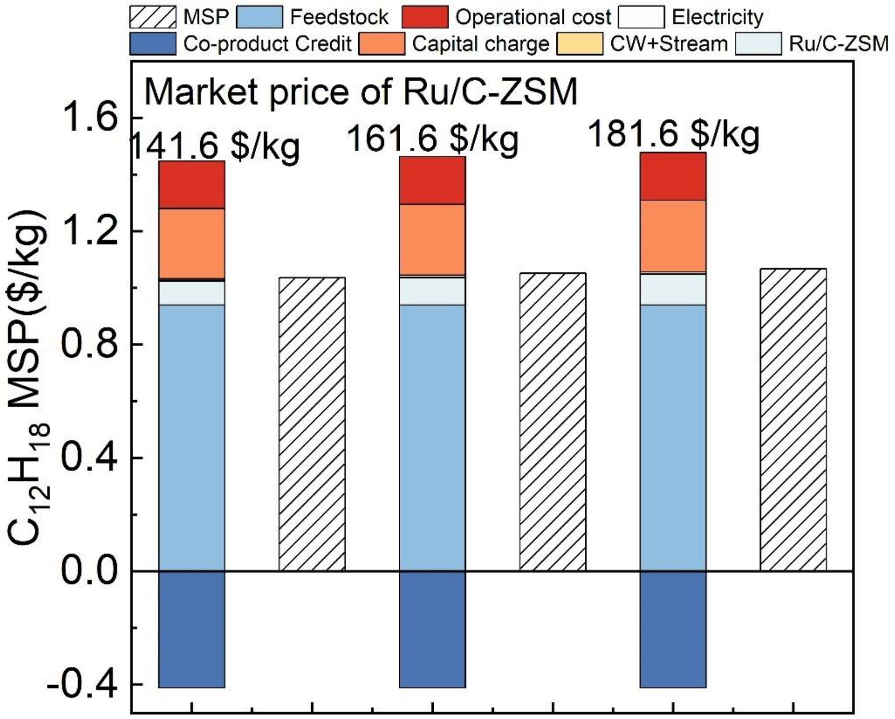

Supplementary Figure 39. Cost breakdown of the $rC_{12}H_{18}$ MSP in the base case process design and as a function of Ru/C-ZSM-5 price.

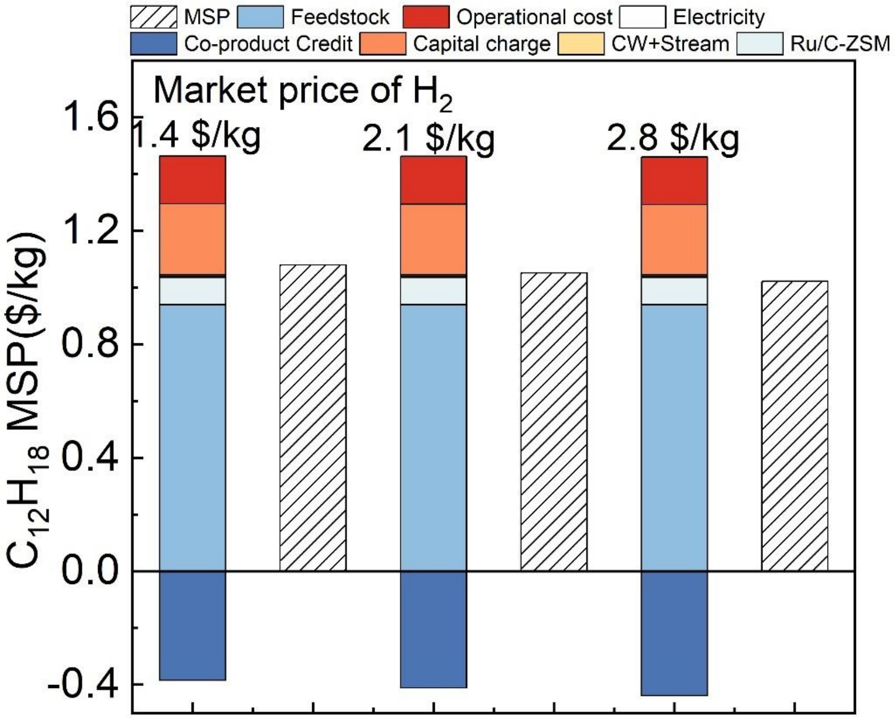

Supplementary Figure 40. Cost breakdown of the $rC_{12}H_{18}$ MSP in the base case process design and as a function of H_2 price.

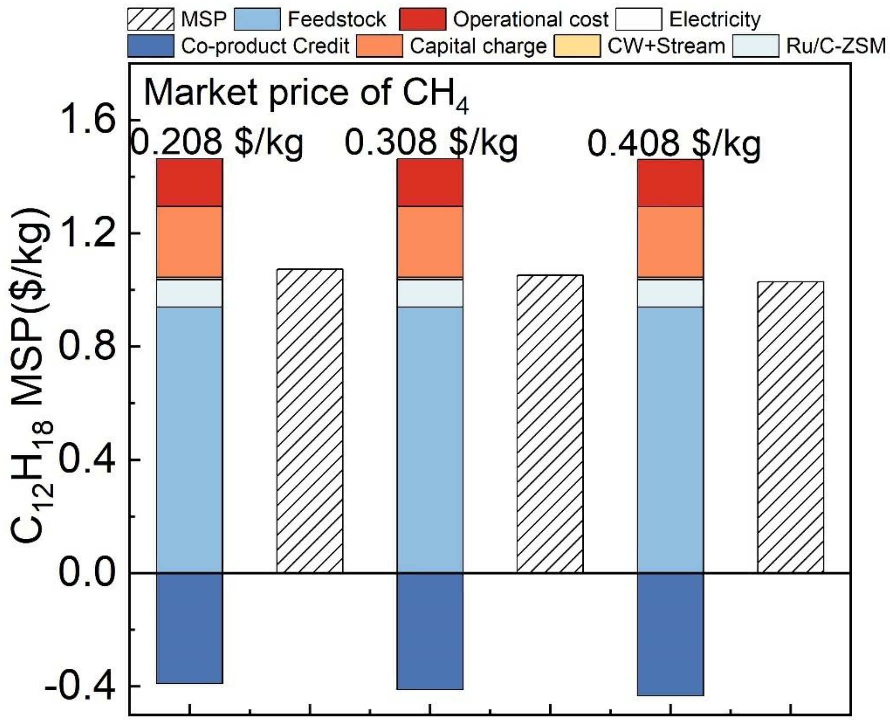

Supplementary Figure 41. Cost breakdown of the $rC_{12}H_{18}$ MSP in the base case process design and as a function of CH_4 price.

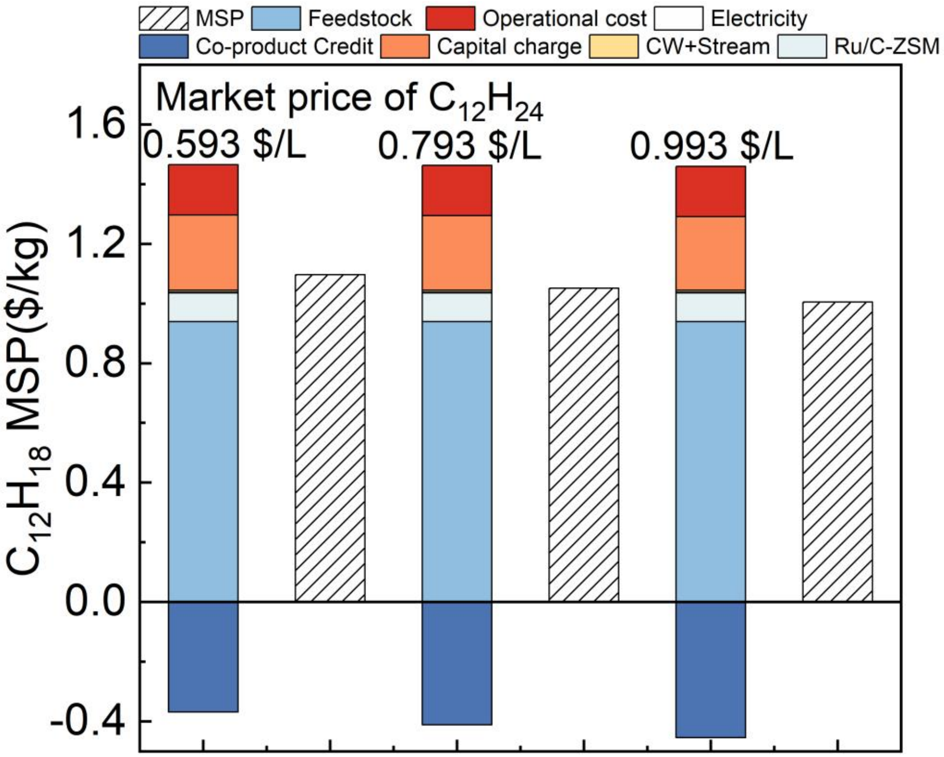

Supplementary Figure 42. Cost breakdown of the r$C_{12}H_{18}$ MSP in the base case process design and as a function of $C_{12}H_{24}$ price.

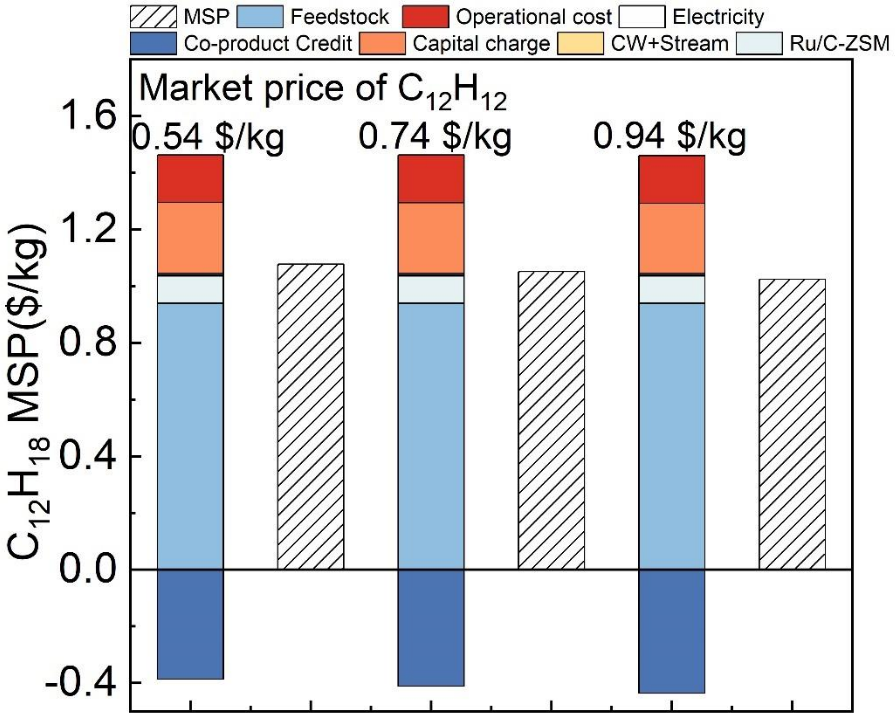

Supplementary Figure 43. Cost breakdown of the r$C_{12}H_{18}$ MSP in the base case process design and as a function of $C_{12}H_{12}$ price.

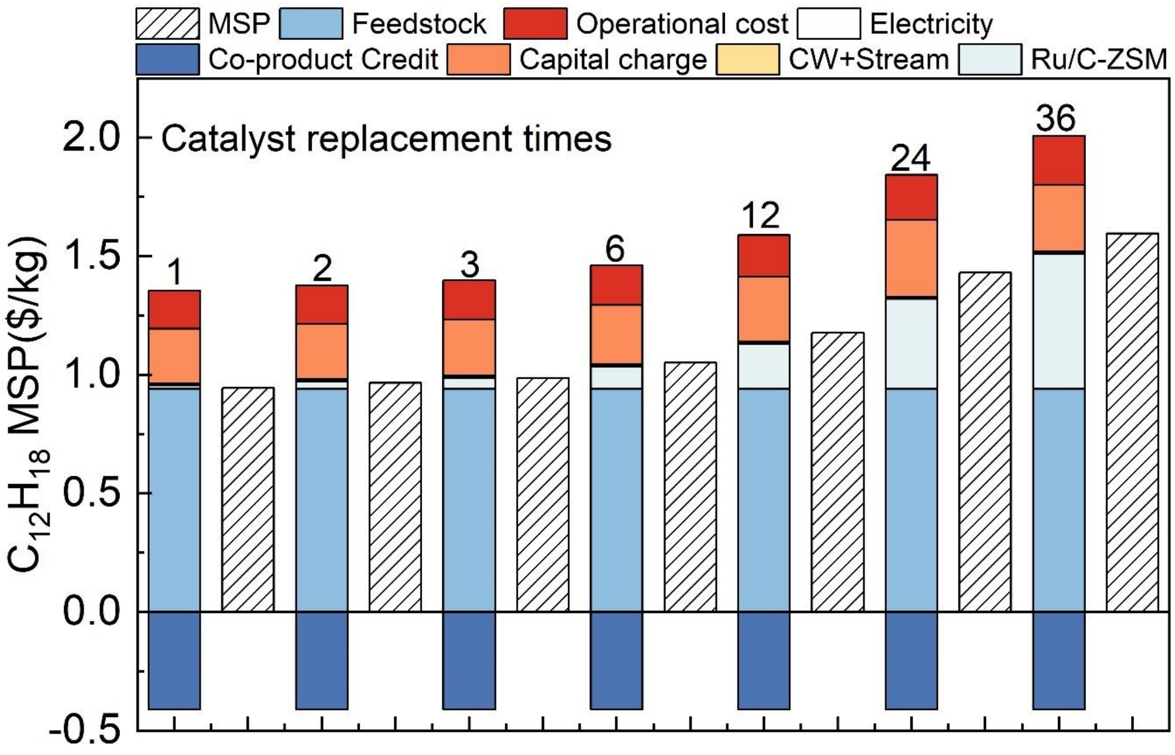

Supplementary Figure 44. Cost breakdown of the r$C_{12}H_{18}$ MSP in the base case process design and as a function of catalyst replacement frequency.

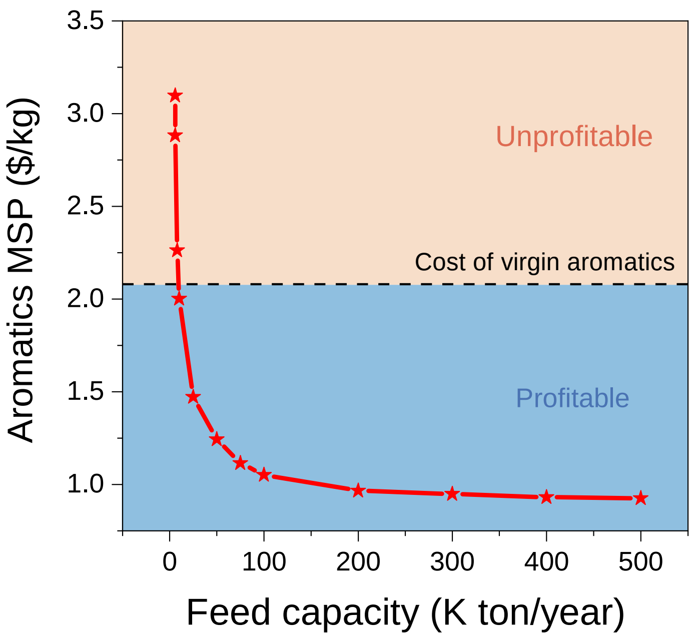

Supplementary Figure 45. Summarization of aromatics MSP as a function of process variables of feed capacity per year.

Table S1. GPC data of liquid products in air and nitrogen for two hours. Mobile phase: tetrahydrofuran

	M_n	M_w
Pristine PE	1694	4582
After 2 h in air	168	185
After 2 h in N_2	176	190

Table S2. Quantification of the acidity of different catalysts by pyridine infrared

Catalyst	Temperature (°C)	B-acid concentration (μmol g^{-1})	L-acid concentration (μmol g^{-1})	Total acid concentration (μmol g^{-1})
ZSM-5	200	160.80	143.59	**340.39**
(Si/Al=70)	350	131.74	39.63	**170.38**
ZSM-5	200	11.79	96.02	**107.81**
(Si/Al=170)	350	2.64	11.21	**13.85**
ZSM-5	200	5.53	65.37	**70.91**
(Si/Al=350)	350	1.67	9.07	**10.74**
Ru/ZSM-5	200	11.71	47.26	**58.98**
(Si/Al=170)	350	6.79	18.64	**25.44**

Compared with pristine ZSM-5 sample (Si/Al = 170), the total acidity of the supported catalyst at 200 °C decreases from 107.8 μmol g^{-1} to 58.9 μmol g^{-1}, and at 350 °C, increases from 25.4 μmol g^{-1} to 13.8 μmol g^{-1}.

Table S3. Catalytic activity of PE for with varying reaction conditions in air.

Temperature	Time	Conversion %	STSC $mg_{PE}\ g_{cat}^{-1}\ h^{-1}$
220 ºC	1	**24.1**	**964.0**
220 ºC	2	**33.6**	**672.0**
220 ºC	4	**36.0**	**360.0**
220 ºC	12	**42.3**	**141.0**
250 ºC	1	**67.0**	**2680.0**
250 ºC	2	**70.0**	**1400.0**
250 ºC	4	**83.0**	**830.0**
250 ºC	12	**91.1**	**303.6**
280 ºC	1	**68.5**	**2740.0**
280 ºC	2	**72.0**	**1440.0**
280 ºC	4	**85.1**	**851.0**
280 ºC	12	**92.3**	**307.6**

Catalytic conditions: 200 mg ZSM-5 (170), 50 mg Ru/C, 1 g PE with ambient air.

Table S4. Benson group increments for various sub-groups in linear polyethylene.

Group label	Chemical identity	$\Delta_f H^{\circ}_{gas,est}$(kJ mol^{-1})	$S^{o}_{gas,est}$(J mol^{-1} K^{-1})
g_1	$-CH_2-$	-21.0	39.4
g_2	$-CH_3$	-42.7	127.2
g_3	CH_2-C=C	-20.0	41.0
g_4	C_{Ar}-H	14.0	48.2
g_5	C_{Ar}-C	23.0	-32.1
g_6	Ar-$C_{\alpha}H_2$-	-20.0	39.1
g_7	Ring strain	3.0	-25.0
g_8	H_2	0	130.7
g_9	O_2	0	205.2
g_{10}	H_2O(g)	-241.8	188.8

Table S5. Ru content in Ru/C catalyst before and after reaction.

	**Ru quality score (wt%)**
Before	4.9
After	4.7

Table S6. Quantitative analysis of catalyst acidity before and after reaction by pyridine infrared.

Catalyst	Temperature (ºC)	B-acid amount (μmol g^{-1})	L-acid amount (μmol g^{-1})	Total acid amount (μmol g^{-1})
ZSM-5	200	11.79	96.02	**107.81**
(Before Cycle)	350	2.64	11.21	**13.85**
ZSM-5	200	14.56	92.14	**106.71**
(After 5 Cycles)	350	5.17	25.19	**30.36**

Table S7. Detailed PDF of PE recovery process section (base case).

Component	Unit	S1	S2	S3	S4	S5	S6	S7	S8	S9
**Total Stream**	kg/hr	12500	135.171	18885.2	18885.2	652.57	18232.6	344.32	308.25	1855.73
**Temperature**	℃	25	25	280	25	25	25	25	25	25
**PE**	kg/hr	12500	135.171	-	-	-	-	-	-	-
**O_2**	kg/hr	-	-	0.264	0.264	0.256	0.0076	0.2565	-	0.0076
**RU/C-ZSM**	kg/hr	-	-	6250	6250	-	6250	-	-	-
**CO_2**	kg/hr	-	-	127.4	127.4	93.38	34.0087	93.38	-	33.998
**CO**	kg/hr	-	-	74.0275	74.0275	72.18	1.849	72.18	-	1.849
**H_2**	kg/hr	-	-	308.752	308.752	308.25	0.5009	-	308.25	0.5
**$C_{12}H_{18}$**	kg/hr	-	-	8066.83	8066.83	0.075	8066.75	0.075	-	85.52
**$C_{12}H_{12}$**	kg/hr	-	-	1002.72	1002.72	0.00077	1002.72	0.00077	-	0.888
**C_4H_{10}**	kg/hr	-	-	1894.55	1894.55	178.40	1716.14	178.4	-	1701.98
**$C_{11}H_{24}$**	kg/hr	-	-	1160.64	1160.64	0.02754	1160.61	0.02753	-	30.985

Component	Unit	S10	S11	S12	S13	S14	S15	S16	S17	S18
**Total Stream**	kg/hr	16376.87	6250	1701.98	153.75	10126.9	9127.5	999.367	1148.08	7979.43
**Temperature**	℃	25	25	25	25	25	197	272	130	215
**PE**	kg/hr	-	-	-	-	-	-	-	-	-
**O_2**	kg/hr	1.96E-07	-	-	0.00762	2.0E-07	1.96-07	4.7E-80	2.0E-07	1.1E-77
**RU/C-ZSM**	kg/hr	6250	6250	-	-	-	-	-	-	-
**CO_2**	kg/hr	0.011	-	-	33.998	0.011	0.011	2.7E-67	0.011	3.58E-64
**CO**	kg/hr	4.1-05 7	-	-	1.849	4.1E-05	4.1E-05	7.8E-78	4.1E-05	1.64E-75
**H_2**	kg/hr	7.0E-07	-	-	0.5	7.E-07	7.E-07	5.8E-95	7. E-07	3.97E-93
**$C_{12}H_{18}$**	kg/hr	7981.23	-	-	85.52	7981.23	7981.23	1.00088	8.787	7971.45
**$C_{12}H_{12}$**	kg/hr	1001.83	-	-	0.8884	1001.83	3.4682	998.367	8.1E-14	3.468
**C_4H_{10}**	kg/hr	14.16	-	1701.98	-	14.1635	14.1635	6.7E-43	14.1635	2.532E-39
**$C_{11}H_{24}$**	kg/hr	1129.63	-	-	30.985	1129.63	1129.63	1.3E-05	1125.12	4.5

Table S8. Yearly operating cost breakdown (base case).

Operating Parameters			
Metric/Parameter	Value	Units	
Annual operating factor	8000	hrs/yr	
Feedstock contaminants	5%	Wt%	
Feedstock PE	100000	Tonnes/yr	
Total $C_{12}H_{18}$ rate	63771.6	Tonnes/yr	
$C_{12}H_{18}$ yield	0.64	Tonnes $C_{12}H_{18}$/tonne PE feed	
PE flake feed mass flow	12500	Kg/h	
$C_{12}H_{18}$ production rate	7971.45	Kg/h	
Variable Operating Costs			
Process hierarchy	Raw material/utility	Mass flow,kg/h	$M/yr
Raw materials			
Feedstock pretreatment	PE flake feedstock	12500	60
PE depolymerization	Ru/C-ZSM	6250/time	6.06
Clarification	Ultrafiltration unit replacement	-	0.28
Crystallization	Membrane replacement	-	0.02
OSBL utilities	HP steam	-	0.3545
	Cooling water	-	0.0188
	Chiller water	-	
	Grid electricity	-	0.204
	Subtotal		66.9373
Co-products and credits			
Filter	H_2 co-product	308.251	5.1786
Distillation	C_4H_{10} co-product	1701.981	4.1937
	$C_{12}H_{12}$ co-product	998.367	5.9103
	$C_{12}H_{24}$ co-product	1125.116	10.9282
	Subtotal		26.2108
Total variable operating cost			**40.7265**
Fixed operating costs			
Labor & supervision			
Total salaries(managers, supervisors, engineers, technicians, administrative staff)			2.16
Labor burden(90% of total salaries)			1.944
Other overhead			
maintenance			0.612
Property insurance & tax			5.7067
Total fixed operating costs			**10.4227**
Total operating costs			**51.1492**

Table S9. Simplified breakdown of the minimum selling price of r$C_{12}H_{18}$ in the base case.

**Cost Category**	**Cost Contribution ($/kg** r$C_{12}H_{18}$**)**
Feedstock	0.9409
Ru/C-ZSM	0.0950
Electricity	0.0032
CW + Stream	0.0059
Capital charge	0.2497
Operational cost	0.1681
Co-product Credit	-0.4110
**MSP**	**1.0518**

Table S10. Waste PE sensitivity results.

Case description	Cost category contribution (**$/kg** $rC_{12}H_{18}$)							MSP (**$/kg** $rC_{12}H_{18}$)
	Feedstock	Ru/C-ZSM	Electricity	CW + Stream	Capital charge	Operational cost	Co-product Credit	
Base case								
Base case	0.94086	0.09503	0.0032	0.00585	0.24976	0.16814	-0.41101	1.05182
Waste PE sensitivities								
Waste PE cost of 0.3 $/kg	0.47043	0.09503	0.0032	0.00585	0.1268	0.13051	-0.41101	0.42081
Waste PE cost of 0.9 $/kg	1.41129	0.09503	0.0032	0.00585	0.28276	0.20567	-0.41101	1.59278

Table S11. Ru/C-ZSM sensitivity results.

Case description	Cost category contribution (**\$/kg** r$C_{12}H_{18}$)							MSP (**\$/kg** r$C_{12}H_{18}$)
	Feedstock	Ru/C-ZSM	Electricity	CW + Stream	Capital charge	Operational cost	Co-product Credit	
**Base case**								
Base case	0.94086	0.09503	0.0032	0.00585	0.24976	0.16814	-0.41101	1.05182
**Waste PE sensitivities**								
Ru/C-ZSM cost of 141.6 \$/kg	0.94086	0.08327	0.0032	0.00585	0.24707	0.16736	-0.41101	1.03659
Ru/C-ZSM cost of 181.6 \$/kg	0.94086	0.10679	0.0032	0.00585	0.25293	0.16893	-0.41101	1.06754

Table S12. H_2 sensitivity results.

Case description	Cost category contribution (**\$/kg** $rC_{12}H_{18}$)							MSP (**\$/kg** $rC_{12}H_{18}$)
	Feedstock	Ru/C-ZSM	Electricity	CW + Stream	Capital charge	Operational cost	Co-product Credit	
Base case								
Base case	0.94086	0.09503	0.0032	0.00585	0.24976	0.16814	-0.41101	1.05182
Waste PE sensitivities								
H_2 cost of 1.4 \$/kg	0.94086	0.09503	0.0032	0.00585	0.25146	0.16814	-0.38394	1.0806
H_2 cost of 2.8 \$/kg	0.94086	0.09503	0.0032	0.00585	0.24829	0.16814	-0.43808	1.02329

Table S13. CH_4 sensitivity results.

Case description	Cost category contribution (**$/kg** $rC_{12}H_{18}$)							MSP (**$/kg** $rC_{12}H_{18}$)
	Feedstock	Ru/C-ZSM	Electricity	CW + Stream	Capital charge	Operational cost	Co-product Credit	
Base case								
Base case	0.94086	0.09503	0.0032	0.00585	0.24976	0.16814	-0.41101	1.05182
Waste PE sensitivities								
CH_4 cost of 0.208 $/kg	0.94086	0.09503	0.0032	0.00585	0.25024	0.16814	-0.38966	1.07366
CH_4 cost of 0.408 $/kg	0.94086	0.09503	0.0032	0.00585	0.24854	0.16814	-0.43236	1.02925

Table S14. $C_{12}H_{24}$ sensitivity results.

Case description	Cost category contribution (**$/kg** $rC_{12}H_{18}$)							MSP (**$/kg** $rC_{12}H_{18}$)
	Feedstock	Ru/C-ZSM	Electricity	CW + Stream	Capital charge	Operational cost	Co-product Credit	
Base case								
Base case	0.94086	0.09503	0.0032	0.00585	0.24976	0.16814	-0.41101	1.05182
Waste PE sensitivities								
$C_{12}H_{24}$ cost of 0.593 $/L	0.94086	0.09503	0.0032	0.00585	0.25244	0.16814	-0.36779	1.09773
$C_{12}H_{24}$ cost of 0.993 $/L	0.94086	0.09503	0.0032	0.00585	0.24658	0.16814	-0.45423	1.00543

Table S15. $C_{12}H_{12}$ sensitivity results.

Case description	Cost category contribution (**$/kg** r$C_{12}H_{18}$)							MSP (**$/kg** r$C_{12}H_{18}$)
	Feedstock	Ru/C-ZSM	Electricity	CW + Stream	Capital charge	Operational cost	Co-product Credit	
Base case								
Base case	0.94086	0.09503	0.0032	0.00585	0.24976	0.16814	-0.41101	1.05182
Waste PE sensitivities								
$C_{12}H_{12}$ cost of 0.54 $/kg	0.94086	0.09503	0.0032	0.00585	0.25073	0.16814	-0.38596	1.07785
$C_{12}H_{12}$ cost of 0.94 $/kg	0.94086	0.09503	0.0032	0.00585	0.24805	0.16814	-0.43606	1.02507

Table S16. Number of catalysts sensitivity results.

Case description	Cost category contribution (**$/kg** $rC_{12}H_{18}$)							MSP **$/kg** $rC_{12}H_{18}$)
	Feedstock	Ru/C-ZSM	Electricity	CW + Stream	Capital charge	Operational cost	Co-product Credit	
Base case								
Base case	0.94086	0.09503	0.0032	0.00585	0.24976	0.16814	-0.41101	1.05182
Number of catalysts sensitivities								
Replaced one time per year	0.94086	0.01584	0.0032	0.00585	0.22901	0.16109	-0.41101	0.94483
Replaced two time per year	0.94086	0.03168	0.0032	0.00585	0.23316	0.1625	-0.41101	0.96623
Replaced three times per year	0.94086	0.04751	0.0032	0.00585	0.23706	0.16391	-0.41101	0.98739
Replaced twelve times per year	0.94086	0.19005	0.0032	0.00585	0.27412	0.17504	-0.41101	1.17811
Replaced twenty-four times per year	0.94086	0.38011	0.0032	0.00585	0.32273	0.19041	-0.41101	1.43214
Replaced thirty-six times per year	0.94086	0.57016	0.0032	0.00585	0.28143	0.20609	-0.41101	1.59658

Table S17. Plant sensitivity results.

Case description	Cost category contribution (**$/kg** $rC_{12}H_{18}$)							MSP (**$/kg** $rC_{12}H_{18}$)
	Feedstock	Ru/C-ZSM	Electricity	CW + Stream	Capital charge	Operational cost	Co-product Credit	
Base case								
Base case	0.94086	0.09503	0.0032	0.00585	0.24976	0.16814	-0.41101	1.05182
Plant sensitivities								
Plant size of 5,800 ton per year	0.94086	0.09503	0.05334	0.00587	0.93821	1.47531	-0.41098	3.09764
Plant size of 6,000 ton per year	0.92973	0.0939	0.05095	0.00579	0.7966	1.41131	-0.40614	2.88215
Plant size of 8,000 ton per year	0.94086	0.09503	0.03869	0.00586	0.5023	1.0902	-0.411	2.26194
Plant size of 10,000 ton per year	0.94086	0.09503	0.03097	0.00585	0.45437	0.88585	-0.41101	2.00191
Plant size of 25,000 ton per year	0.94086	0.09503	0.01248	0.00585	0.41832	0.40945	-0.41101	1.47097
Plant size of 50,000 ton per year	0.94086	0.09503	0.00629	0.00585	0.35896	0.24753	-0.41101	1.24351
Plant size of 75,000 ton per year	0.94086	0.09503	0.00423	0.00585	0.28666	0.19356	-0.41101	1.11518
Plant size of 200,000 ton per year	0.94086	0.09503	0.00165	0.00585	0.2075	0.12662	-0.41101	0.9665
Plant size of 300,000 ton per year	0.94086	0.09503	0.00114	0.00585	0.20097	0.11522	-0.41101	0.94805
Plant size of 400,000 ton per year	0.94086	0.09503	8.80878E-4	0.00585	0.19139	0.1086	-0.41101	0.9316
Plant size of 500,000 ton per year	0.94086	0.09503	7.26029E-4	0.00585	0.18543	0.10902	-0.41101	0.92591

Table S18. Univariate sensitivity summary.

Sensitivity	Sensitivity parameter range				Bound MSP (**$/kg** r$C_{12}H_{18}$)		% MSP Difference (from base case)	
	Units	Lower MSP case	Base case	Higher MSP case	Lower MSP	Higher MSP	Lower %	Upper %
Plant size	K ton/year	500	100	5.8	0.92591	3.09764	-11.971	194.502
Feedstock cost	$/kg	0.3	0.6	0.9	0.42081	1.59278	-59.993	51.43
Number of catalysts	/time	1	6	36	0.94483	1.59658	-10.172	51.791
$C_{11}H_{24}$ price	$/L	0.993	0.793	0.593	1.00543	1.09773	-4.411	4.364
H_2 price	$/kg	2.8	2.1	1.4	1.02329	1.0806	-2.713	2.736
$C_{12}H_{12}$ price	$/kg	0.94	0.74	0.54	1.02507	1.07785	-2.544	2.474
CH_4 price	$/kg	0.408	0.308	0.208	1.02925	1.07366	-2.146	2.076
Ru/C-ZSM price	$/kg	141.6	161.6	181.6	1.03659	1.06754	-1.448	1.494

References

1. Zhang, F. *et al.* Polyethylene upcycling to long-chain alkylaromatics by tandem hydrogenolysis/aromatization. *Science* **370**, 437–441 (2020).
2. Chen, W. *et al.* One-pot upgrading polyethylene and CO_2 to aromatics via tandem catalysis. *CCS Chem.* **6**, 1–23 (2023).
3. Sun, J. *et al.* Bifunctional tandem catalytic upcycling of polyethylene to surfactant-range alkylaromatics. *Chem* **9**, 2318–2336 (2023).
4. Scheffé, H. Experiments with Mixtures. *Journal of the Royal Statistical Society Series B: Statistical Methodology* **20**, 344–360 (1958).
5. Scheffé, H. The Simplex-Centroid Design for Experiments with Mixtures. *Journal of the Royal Statistical Society Series B: Statistical Methodology* **25**, 235–251 (1963).
6. Behrens, J. T. Principles and procedures of exploratory data analysis. *Psychological Methods* **2**, 131–160 (1997).
7. Stuart, M. Review of Understanding Robust and Exploratory Data Analysis. *Journal of the Royal Statistical Society. Series D (The Statistician)* **33**, 320–321 (1984).
8. Jing, Y. *et al.* Towards the Circular Economy: Converting Aromatic Plastic Waste Back to Arenes over a Ru/Nb_2O_5 Catalyst. *Angew. Chem. Int. Ed.* **60**, 5527–5535 (2021).
